# Deciphering the impact of aging on splenic endothelial cell heterogeneity and immunosenescence through single-cell RNA sequencing analysis

**DOI:** 10.1186/s12979-024-00452-1

**Published:** 2024-07-18

**Authors:** Yanjing Huang, Zhong Liu, Mengke Li, Dongliang Wang, Jinguo Ye, Qiuling Hu, Qikai Zhang, Yuheng Lin, Rongxin Chen, Xuanwei Liang, Xingyi Li, Xianchai Lin

**Affiliations:** State Key Laboratory of Ophthalmology, Zhongshan Ophthalmic Center, Guangdong Provincial Key Laboratory of Ophthalmology and Visual Science, Sun Yat-sen University, Guangzhou, 510060 China

**Keywords:** scRNA-seq, Spleen, Aging, Endothelial cell, Immunosenescence

## Abstract

**Background:**

Aging is associated with significant structural and functional changes in the spleen, leading to immunosenescence, yet the detailed effects on splenic vascular endothelial cells (ECs) and their immunomodulatory roles are not fully understood. In this study, a single-cell RNA (scRNA) atlas of EC transcriptomes from young and aged mouse spleens was constructed to reveal age-related molecular changes, including increased inflammation and reduced vascular development and also the potential interaction between splenic endothelial cells and immune cells.

**Results:**

Ten clusters of splenic endothelial cells were identified. DEGs analysis across different EC clusters revealed the molecular changes with aging, showing the increase in the overall inflammatory microenvironment and the loss in vascular development function of aged ECs. Notably, four EC clusters with immunological functions were identified, suggesting an Endothelial-to-Immune-like Cell Transition (EndICLT) potentially driven by aging. Pseudotime analysis of the Immunology4 cluster further indicated a possible aging-induced transitional state, potentially initiated by Ctss gene activation. Finally, the effects of aging on cell signaling communication between different EC clusters and immune cells were analyzed.

**Conclusions:**

This comprehensive atlas elucidates the complex interplay between ECs and immune cells in the aging spleen, offering new insights into endothelial heterogeneity, reprogramming, and the mechanisms of immunosenescence.

**Supplementary Information:**

The online version contains supplementary material available at 10.1186/s12979-024-00452-1.

## Background

The spleen, a secondary lymphoid organ, is integral to the initiation of immune responses to blood-borne antigens [1]. Its pivotal role extends beyond systemic immunity, encompassing both innate and adaptive immune systems, and includes hematopoiesis in childhood or severe blood loss [2]. However, the aging process imposes significant alterations on the spleen’s structure and function [3, 4]. Aging is associated with an enlargement of the spleen’s white pulp, particularly within the T-cell zone, and a corresponding expansion of the T-cell stromal area. This is accompanied by a blurring of the distinct boundary between T cells and B cells in older mice [5, 6]. These structural modifications impact the functionality of the resident immune cells, potentially leading to diminished or less effective immune responses [7]. Aging also lead to immune dysfunction in spleen, causing a gradual deterioration of immune systems, a process known as Immunosenescence [4]. This manifests as a reduced capacity to mount effective responses to pathogens and cancer cells [4].

Moreover, the spleen, being a highly vascularized organ, experiences profound effects of aging on its vascular endothelial cells, including endothelial dysfunction and an increase in inflammatory signaling [8, 9]. Recent studies have confirmed that subsets of ECs in various tissues and organs possess immunomodulatory activities beyond their established role in alloimmunity, immune cell recruitment, immune tolerance, and vascular inflammation [10–12]. However, the specific impacts of aging on different types of splenic ECs and its effects on their immunomodulatory function remain largely unexplored.

In this study, we provide a comprehensive transcriptional atlas derived from single-cell RNA sequencing (scRNA-seq) of young and old mouse splenic endothelial cells. Our findings present a detailed portrayal of the changes in cellular and molecular complexity associated with aging at a single-cell resolution.

This atlas allows us to investigate the effects of aging on gene expression across different subsets, to illustrate unique biological processes that emerge in the aging vascular endothelium, and to explore potential interactions between splenic ECs and spleen immune cells. Overall, our research enhances the understanding of the aging process in splenic ECs and provides valuable insights into the interplay between splenic ECs and immune cells.

## Methods

### Animals

The 6–8-week and 24-month wild-type C57BL6/J mice have been housed since birth in the Animal Management Center at Zhongshan Ophthalmic Center. All animal experimentation procedures in compliance with the laws governing animal research. The young and old mice were processed parallelly, including all baseline information, feeding environment, material retrieval method, quality control of sequencing data, and other pertinent details.

### Spleen ECs collection

After perfusion with PBS, the mouse spleens were surgically removed, rinsed with ice-cold PBS and dissected into small pieces using sterile scalpels. Then, the tissues were transferred to 5 ml HBSS digestion buffer, including 0.1% collagenase II (Thermo Fisher Scientific, #17,101,015), 0.25% collagenase IV (Thermo Fisher Scientific, #17,104,019), 2 mg/mL DNase I (Sigma-Aldrich, #11,284,932,001), and incubated in a 37 ◦C water bath for 15 min, pipetting it with a 1 ml tip every 3 min. 1 ml of 10% FBS (Thermo Fisher Scientific, #A5669701) was used to terminate digestion. The cell suspension is filtered through a 40 mm cell strainer (Sigma-Aldrich, #CLS431750-50 EA) and centrifuged at 300 g for 5 min. Carefully remove the obtained supernatant and then enrich the ECs in the cell suspension using CD31 microbeads (Miltenyi Biotec, #130-097-418) in DMEM medium containing 10% FBS (GIBCO, #C11330500BT) according to the manufacturer’s instructions.

### Library preparation and sequencing

We resuspended freshly isolated splenic ECs in PBS containing 0.04% ultra-pure BSA. According to the manufacturer’s instructions, scRNA-seq libraries were prepared using the Chromium Single Cell 3’ Reagent Kits v2 (10x Genomics; Pleasanton, CA, USA). All libraries were aimed at a target recovery of 5,000 cells. The generated libraries were sequenced on an Illumina HiSeq 4000, followed by demultiplexing and mapping to the mouse genome (build mm10) using CellRanger (10x Genomics, version 2.1.1).

### Quality control of scRNA-seq data

Using the CellRanger software (10x Genomics), gene expression matrices were generated. Sample data was aggregated using the CellRanger software and raw data was analyzed in Seurat R package (version 4.3.1). The lognormalization of the data of the young and old splenic EC samples was performed using Seurat’s “LogNormalize” algorithm. With “FindIntegrationAnchors”, we selected features and anchors for downstream integration, making sure that all cells were calculated. Then the following quality control steps were performed:

(i) genes expressed by less than 3 cells or with a row average of < 0.002 were not considered.
(ii) cells that expressed fewer than 200 genes (low quality), and cells that expressed over 4,000 genes (potential doublets) were excluded from further analysis.
(iii) cells in which over 10% of unique molecular identifiers (UMIs) were derived from the mitochondrial genome were removed.

### Clustering and identification of cell types

After data integration and scaling, the principal component analysis was performed by the “RunPCA” function. Then the graph-based clustering was performed to cluster cells according to their gene expression profile using the “FindClusters” function in Seurat (clustering resolution = 0.3, k-nearest neighbors = 20). Marker genes for each cluster were identified with the “FindAllMarkers” function, and only those min.pct > 0.25, logfc.threshold > 0.25 were considered as markers genes. We screened the top 50 of these ranked marker gene lists for each identified cluster for coherent enrichment of known canonical marker genes of traditional EC subtypes (i.e., Artery, Capillary arterial, Capillary venous), as well as genes involved in particular cellular pathways or processes, to facilitate putative annotation of the clusters (i.e. Immunology1, Immunology2, Proliferating) according to a biologically meaningful phenotype.10 shared clusters were annotated in young and old splenic ECs. The spearman correlation analysis is performed by “AvergeExpression” function. (Fig. 3C, Fig.S1E)

### Immunofluorescence staining

We performed immunofluorescence staining as previously described [13].

#### (1) Tissue fixation

After washing the tissues thoroughly, they were immediately placed into fixation solution (prepared with DEPC water) and fixed for over 12 h.

#### (2) Dewaxing and dehydration

Immerse the sections in two consecutive baths of BioDewax and Clear solution for 15 min each. Subsequently, dehydrate them in two rounds of pure ethanol, 5 min each. Then, proceed with dehydration in 85% and 75% ethanol gradients for 5 min each. Finally, rinse in DEPC dilution.

#### (3) Digestion

Depending on the duration of tissue fixation, the slices are boiled in the retrieval solution for 10–15 min and naturally cooled. Subsequently, the target areas are marked using the liquid blocker pen and based on the specific characteristics of different tissues, proteinase K (5ug/ml) is added and incubated at 37 °C for 5 min for digestion. After rinsing with pure water, the sections are washed three times with PBS for 5 min each.

#### (4) Block endogenous peroxidase

Addition of 3% methanol-H2O2, incubate at room temperature in the dark for 15 min. Place the slides in PBS (pH 7.4) and shake on a decolorization shaker for 3 washes, each lasting 5 min.

#### (5) Addition of blocking solution

Add blocking serum BSA. Incubate at room temperature for 30 min.

#### (6) Incubation with primary antibody

Add the CD31 primary antibody(GB120005-100,Servicebio) diluted in PBS at a ratio of 1:200. Incubate at 4 °C overnight. Subsequently, wash with PBS 3 times for 5 min each.

#### (7) Incubation with second antibody

Add the corresponding secondary antibody, Goat Anti-Rabbit AF488(ab150081,abcam) and incubate at room temperature for 50 min. Subsequently, wash with PBS 3 times for 5 min each.

#### (8) Add cy3-TSA

Add cy3-TSA reagent and incubate in the dark at room temperature for 5 min. Subsequently, wash with PBS 3 times for 5 min each.

#### (9) Pre-hybridization

Add Pre-hybridization solution to each section and incubate for 1 h at 37℃.

#### (10) Hybridization

Discard the pre-hybridization solution, then add the hybridization solution containing probes for cd14, cd52, cd79a, and fcerla at a concentration of 1 μm. Hybridize overnight at 40 degrees Celsius in an incubator.

#### (11) Washing

Eliminate the hybridization solution. Submerge sections in 2×SSC and wash for 10 min at 37℃. Then, perform two 5-minute washes with 1×SSC at 37℃. Finally, wash sections in 0.5×SSC for 10 min at room temperature. If there are additional non-specific hybrids, consider adding formamide during washing.

#### (12) Blocking

Add blocking solution (Rabbit serum) to the section and incubate at room temperature for 30 min.

#### (13) Add the mouse anti-DIG-HRP

Take out the blocking solution and apply anti-DIG-HRP. Incubate at 37 °C for 50 min, followed by washing the sections in PBS three times for 5 min each.

#### (14) TSA developing

Add fresh prepared TSA chromogenic reagent to marked tissue. Reaction in dark for 5 min at room temperature. Then wash sections in PBS three times for 5 min each.

#### (15) Stain cell nuclei (counter stain)

Incubate with DAPI for 8 min in the dark, and then mounting.

#### (16) Microscopic examination and photography

The slices were observed and images were captured under a Nikon upright fluorescence microscope. For UV excitation, the wavelength range was set between 330 and 380 nm with an emission wavelength of 420 nm, emitting blue light. For FAM (488) green fluorescence, the excitation wavelength was set between 465 and 495 nm with an emission wavelength of 515–555 nm, emitting green light. For CY3 red fluorescence, the excitation wavelength was set between 510 and 560 nm with an emission wavelength of 590 nm, emitting red light.

mRNA FISH probes were purchased from Guangzhou Exons biological technology co.,LTD. and Guangzhou Shinak Biotechnology co.,LTD. The antibodies and probes used for immunofluorescence staining are as follows: Anti-CD31(Thermo Fisher, #PA5-32321,1:500), mus Cd14 mRNA FISH Probe (#TNG_mCD14FP), mus Cd52 mRNA FISH Probe (#007.221040), mus Cd79a mRNA FISH Probe (#007.23320), mus Fcerla mRNA FISH Probe (#007.23321). The secondary antibodies used are as follows: Goat Anti-Rabbit AF488 (Thermo Fisher, #A-11,008, 1:500), Anti-Biotin FITC-Conjugate (Bioss, #BS–0437P-FITC, 1:500) and HRP-Monoclonal Mouse Anti- Digoxin Antibody (Jackson, #200-032-156, 1:500).

### Analysis of DEGs between young and old splenic ECs data

Differential gene expression analysis was performed with the “FindMarkers” function of Seurat between old and young groups using the Wilcox test. Only those with adjusted |logFC| > 0.25 and P-values adjusted < 0.05 were selected as up- and down- regulated DEGs.

### GO term and KEGG pathway analysis

GO enrichment analysis of both marker genes and age-related DEGs was performed by Metascape(https://metascape.org/gp/index.html). Results were visualized with the ggplot2 R package (https://ggplot2.tidyverse.org/)(version 3.3.5). KEGG analysis was performed by clusterProfiler R package (version 4.8.3).

### Gene set score analysis

Each gene set score was calculated by using the Seurat function “AddModuleScore”. Gene sets of Immune-related genes (IRG) and Antigen Processing and Presentation are from *Immport* (https://www.immport.org/) [14]. The gene set of immune cell homing(ICH) is from previous article [15]. The Allergy-related gene set is from AllerGAtlas (http://biokb.ncpsb.org.cn/AllerGAtlas) [16]. The B cell Activation (GO:0042113) and Phagocytosis (GO: 0006909) gene set are from Gene Ontology. The gene sets in the article are summarized in Table S3.

### Single-cell consensus weighted gene co-expression network analysis

Single-cell consensus co-expression analysis was performed by hdWGCNA [17, 18], which is an R package for performing weighted gene co-expression network analysis (https://github.com/smorabit/hdWGCNA) in high dimensional transcriptomics data such as single-cell RNA-seq or spatial transcriptomics. hdWGCNA is used to perform construct co-expression network, compute Module Eigengenes(MEs) and connectivity and the analysis of co-expression module dynamics with pseudotime.

### Pseudotime analysis

The Monocle R packages were used for pseudotime analysis. Genes with highly variable values from the “VariableFeatures” function in the Seurat package were used as ordering genes. DDRTree dimensionality reduction method was applied to construct the trajectory that was plotted in two-dimensional space. And Time differentiation related DEGs were obtain with a cutoff of q value < 1e-4.

### Transcriptional regulatory network analysis

A transcriptional regulatory network analysis was conducted by utilizing pySCENIC workflow [19] (version 1.1.2.2). Cell-type-specific transcription regulatory networks were calculated by using all genes from young and old splenic ECs.

### Cell-cell interaction analysis

Cell-cell interaction analysis was conducted using CellChat R package (version1.6.1) [20]. The analysis of aged and young splenic ECs and immune cell was done separately while the comparison between these two groups was performed by integrating data from both sets of analyses. While interactions were calculated between all identified subclusters, we specifically focused our interpretation and analysis on interactions between immunology-related ECs (Immunology1, Immunology2, Immunology3, Immunology4) and immune cell types (DCs, Mast cells, B cells and Macrophages).

### Global and local alignment of single cell trajectories

Global and local quantitative comparison of expression dynamics within young and old trajectories was conducted by cellAlign [21]. The expression matrix of young and old Immunology4 subset (Capillary1,Capillary2,Immunology4,Macrophage, Proliferating) and its pseudotime spacing was used to input in cellAlign workflow.

## Results

### Single-cell transcriptome reveals age-related variations in splenic endothelial cell clusters

**Fig. 1:**
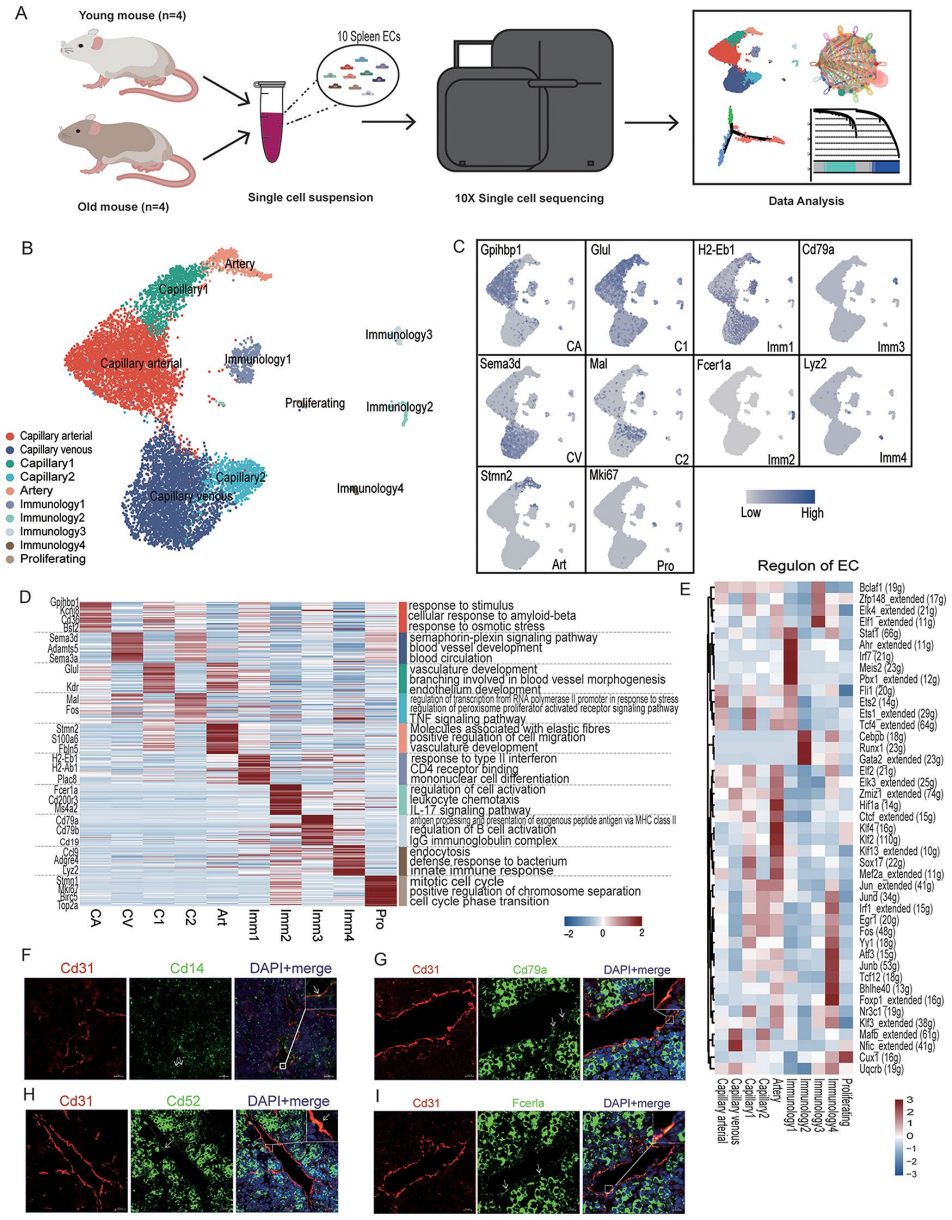
Construction of single cell sequencing atlas of splenic endothelial cells. **(A)** Flow chart of scRNA-seq and bioinformatics analysis of the young and old splenic endothelial cells. (Young, *n* = 5053; Old, *n* = 5414). **(B)** UMAP plot showing different celltypes in mouse splenic endothelial cells. Young and old groups are sharing the same clusters: Capillary arterial, Capillary venous, Capillary1, Capillary2, Artery, Immunology1, Immunology2, Immunology3, Immunology4, Proliferating. **(C)** Feature plots display the expression profiles of celltype-specific marker genes for different clusters in mouse splenic ECs. **(D)** Heatmap showing the top 30 marker genes of specific clusters in the old group and the enrichment function annotations of each are on the right. **(E)** Heatmap showing the particular regulon in different clusters of splenic ECs.**(F-I)** Representative micrographs of old mouse splenic sections, stained for an EC marker (Cd31) and Cd14(Monocytes, F), Cd79a (Immunology3, G), Cd52 (Immunology, H), Fcerla (Mast cell, I) and counterstained with DAPI

To elucidate the transcriptional changes associated with aging in mouse splenic endothelial cells, we isolated ECs from mouse spleen tissues from 4 young (2-month-old, female) and 4 old (2-year-old, female) C57/B16 mice. We barcoded and sequenced the ECs using 10x Genomics-based single-tube protocol and excluded pericytes (Pdgfrb) or immune cells (Ptprc), smooth muscle cells (Acta2), fibroblasts (Col1a1), and erythrocytes (Hba-a1, Hba-a2, Hbb-bs) as per previous analyses [22] and EC markers (Pecam1) have been selected. A total of 10,467 ECs (Young: 5,053, Old: 5,414) were prepared for downstream analysis after strict quality control, doublets checking (Fig. S4) and gene expression filtering (Fig. 1A). Following normalization, unsupervised graph-based clustering partitioned the cells into groups as visualized by the uniform manifold approximation and projection (UMAP) plots, including the clusters UMAP plots (Fig. 1B) and the young and old group UMAP plots (Fig. S1 A).

Apart from the conventional clusters of splenic ECs identified in previous study [22], such as Capillary arterial (Gpihbp1), Capillary venous (Sema3d), Capillary1 (Glul), Capillary2 (Mal), and Artery (Stmn2) (Fig. 1C), we identified 5 additional clusters in both young and old ECs. One of these, the Proliferating EC, which highly expressed the marker Mki67, has been associated with vascular EC regeneration in previous studies [22, 23]. Notably, our cluster marker investigation revealed four distinct clusters of ECs with immunological relevance, as many of their markers are related to immune genes. We conducted an overlap analysis of the top 100 markers of these immunology-related ECs and classic immune cells (Fig. 3B). The results revealed that each subtype of immunology-related ECs displayed a strong association with specific types of immune cells: Immunology1 with dendritic cells (DC), Immunology2 with Mast cell, Immunology3 with B cells, and Immunology4 with Macrophages. They were subsequently named Immunology1 (H2-Eb1), Immunology2 (Fcerla), Immunology3 (Cd79a), and Immunology4 (Lyz2) (Fig. 1C).

We identified a set of Top50 markers for each cell type (Table S1). The cell proportions of different clusters in the young and old splenic groups (Fig. S1 A) indicated that the number of Capillary Venous ECs increase with aging, which shares the same situation in the previous study [23]. Other clusters such as Capillary1, Capillary2 and Capillary Arterial are declined with aging.

Gene Ontology (GO) analysis of the Top 30 marker genes unveiled the functional characteristics linked to each specific cell type (Fig. 1D). The function “branching involved in blood vessel morphogenesis” was enriched for Capillary1, indicating its involvement in the development of vessel branches. For Capiilary2, the function “regulation of transcription from RNA polymerase II promoter in response to stress” was enriched. In addition, the Capillary Arterial, Capillary venous and Artery clusters were highly associate with vascular development. The Proliferating ECs showed a strong ability to regulate the mitotic cell cycle, confirming its role in vascular proliferation.

We further investigated the molecular mechanisms underlying EC phenotypic differentiation using single-cell regulatory network inference and clustering (SCENIC) [19]. We analyzed the regulons exerting the most significant impact on each type of EC (Fig. 1E), elucidating the regulation strength of each regulon for different cell types. Double immunostaining of IF and FISH for an EC marker (CD31) and the markers of these specialized immunology EC phenotypes validated the scRNA-seq data (Fig. 1F–I).

### Age-related cellular and molecular characteristics of murine splenic ECs

**Fig. 2:**
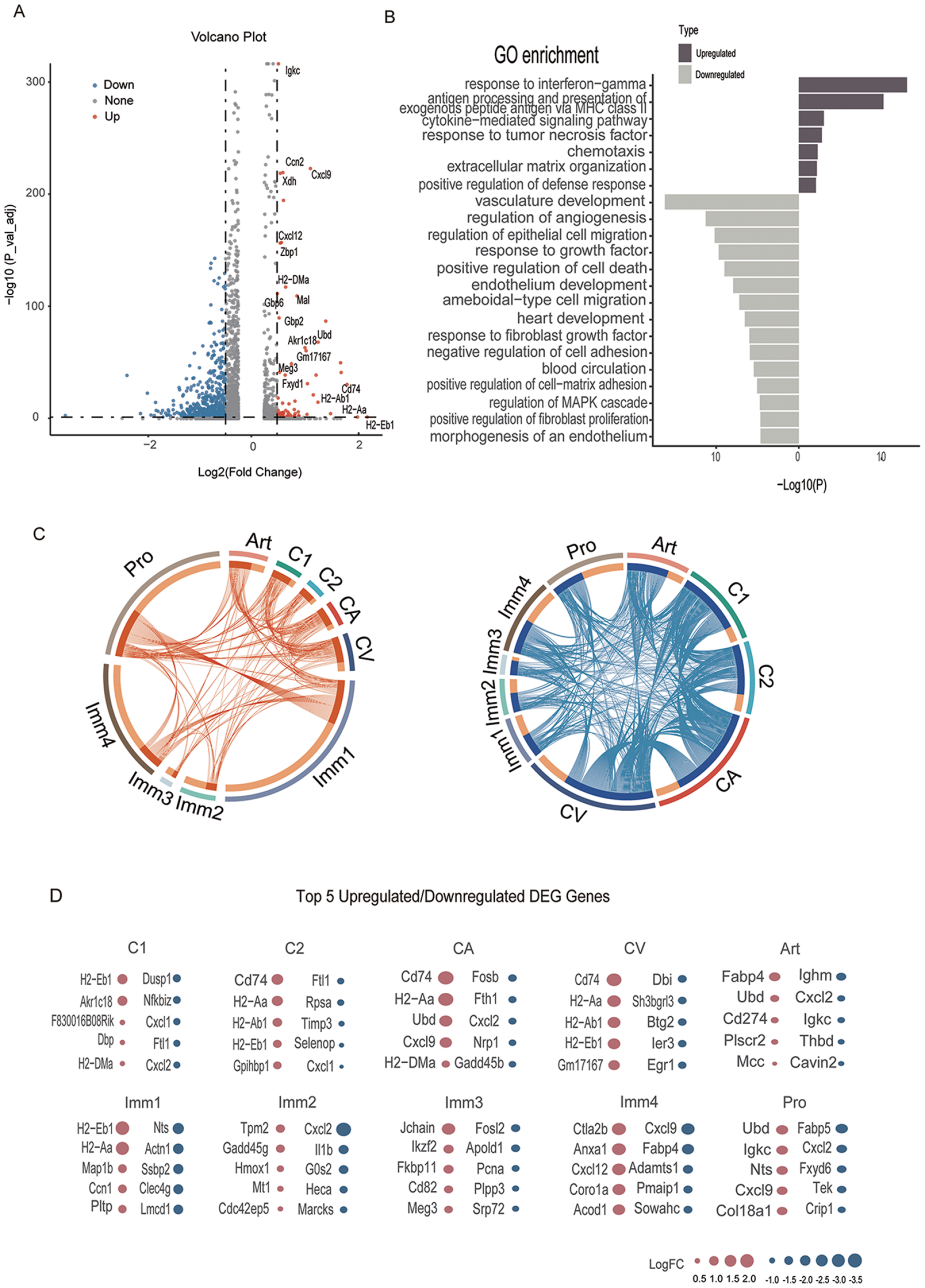
Cellular and molecular characteristics of aged splenic ECs. **(A)** Volcano plot showing aging-associated up- and down- regulated differentially expressed genes (DEGs) in all celltypes (adjusted P-value < 0.05,|LogFC| >0.25), high expression of up-regulated gene are labeled. **(B)** Barplot showing GO terms of the overall DEGs between young and old group in all celltypes. **(C)** Circos plot showing aging-associated up- and down- regulated DEGs, each of the connecting curve showing the gene is whether up- or down- regulated between two clusters. **(D)** Dot plots showing the top five celltype-specific DEGs of different clusters. Only those with annotations are showed. Up-regulated genes are colored in red while the down-regulated ones are in blue

To further elucidate the impact of aging on the molecular function of the splenic endothelium, we analyzed the overall differentially expressed genes (DEGs) in young and old splenic ECs (Fig. 2A and Table S2). We discovered that functions such as “response to interferon-gamma,” “cytokine-mediated signaling pathway,” and “response to tumor necrosis factor” were enriched for upregulated DEGs, indicating an increased expression of inflammatory signatures and a shift towards a pro-inflammatory phenotype (Fig. 2B). These findings align with previous research [24, 25]. Functions such as “endothelium development,” “response to growth factor,” and “blood circulation” were enriched in downregulated DEGs, suggesting vascular dysfunction in senescent endothelial cells [24].

To further investigate cell type-specific alterations in gene expression, we identified key cell types and molecular mechanisms affected by splenic senescence (Table S2). We observed the highest numbers of both up- and down-regulated DEGs in Immunology1 (up:198, down:109), Immunology4 (up:128, down:174), and Proliferating (up:169, down:187) clusters (Fig. S1 C), suggesting these cell types may be most affected by aging. The chord plot (Fig. 2C) indicated more overlapping DEGs in classic clusters (such as Capillary Arterial, Capillary Venous, Capillary1, Capillary2, and Artery) than in unique clusters like Immunology1, Immunology2, Immunology3, Immunology4, and Proliferating. The top 5 DEGs in different clusters revealed an increased immune signature, as illustrated by the overexpression of H2-Aa, H2-Ab1, H2-Eb1, Cd74 in clusters such as Capillary Arterial, Capillary Venous, Capillary1, and Immunology1 (Fig. 2D, Fig. S1 C). This is consistent with existing literature [26–29]. Cxcl12 was found to be downregulated in almost every cluster of splenic ECs (Fig. S1 D), which could affect the ability of immune cell homing for the splenic ECs [10, 30].

### Cellular and molecular characteristics of immunology-related ECs

**Fig. 3:**
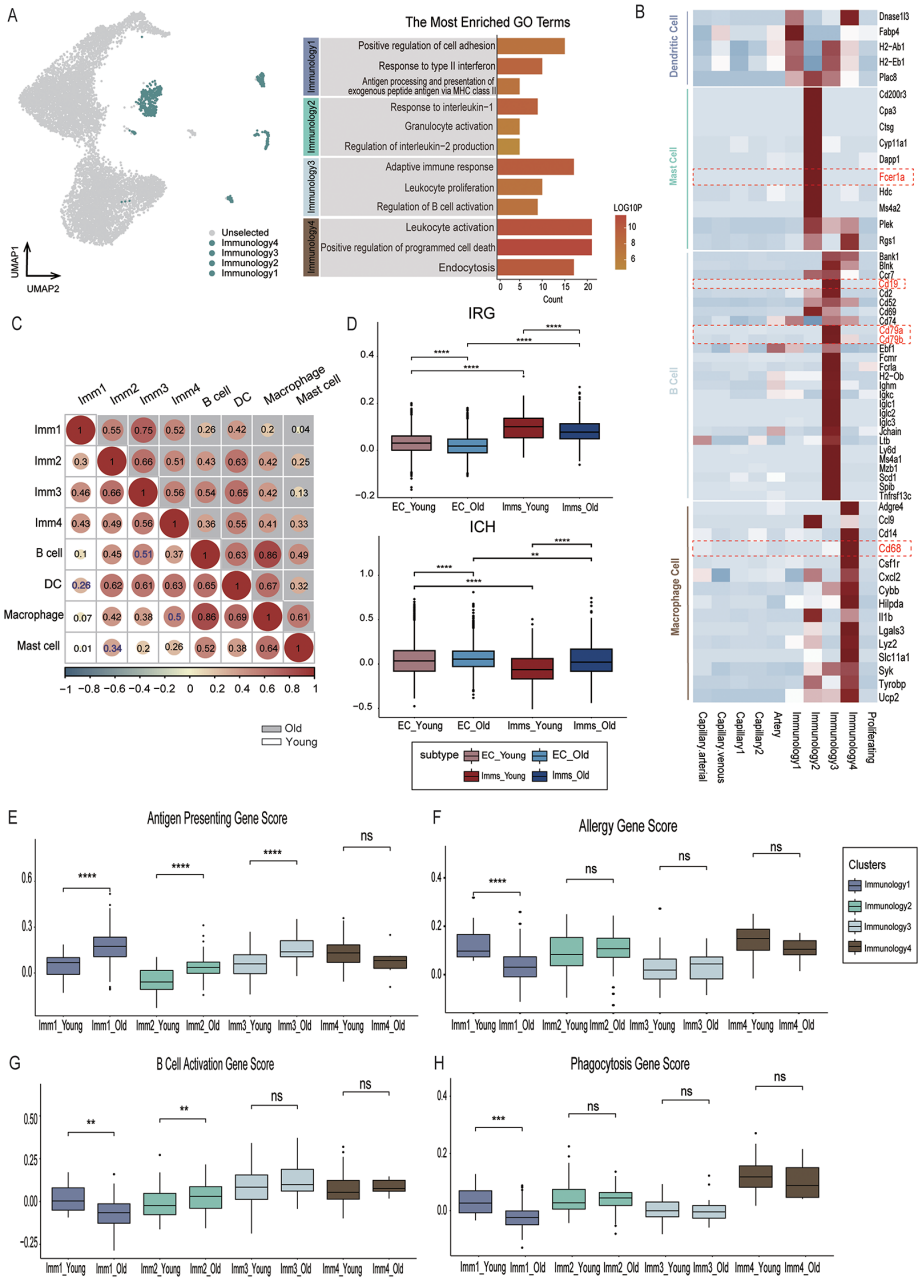
Cellular and molecular characteristics of four immunology-related ECs. **(A)** Left: Feature plots showing the positions of four subtypes of immunology-related ECs. Right: Bar chart illustrating the most enriched Gene Ontology (GO) term within these subtypes. The X-axis represents the number of genes associated with the functions, and the bars are color-coded based on Log10P values. **(B)** Heatmap showing the shared markers between immunology-related ECs and Immune cells (DCs, Mast cells, B cells, Macrophages and monocytes). **(C)** Correlation matrix displaying the relationships between immunology-related ECs and Immune cells. The upper matrix, shaded in grey, illustrates the correlations between Old immunology-related ECs and Immune cells, while the lower matrix depicts the correlations for young ones. Each correlation coefficient is labeled within the corresponding circle in the matrix. **(D)** Upper: Boxplot showing the Immunology-related gene (IRG) score of Young and Old regular ECs subtypes and immunology ECs. ****p < 0.001. Lower: Boxplot showing the immune cell homing (ICH) genes score of Young and Old regular ECs subtypes and immunology ECs. ****p < 0.001. **(E)** Boxplot showing the Antigen Presenting gene score of young and old immunology-related ECs. ****p < 0.001. **(F)** Boxplot showing the Allergy-related gene score of young and old immunology-related ECs. ****p < 0.001. **(G)** Boxplot showing the B cell Activation gene score of young and old immunology-related ECs. ****p < 0.001. **(H)** Boxplot showing the Phagocytosis gene score of young and old immunology-related ECs. ****p < 0.001

We have delineated four distinct subpopulations of ECs with immunological relevance, each demonstrating a strong association with a particular immune cell type (Fig. 3B). Immunology1 cells express dendritic cell markers such as Fabp4 and H2-Eb1, suggesting functional similarities to DC, while Immunology2 cells express Fcerla alongside other mast cell markers, indicating mast cell-like characteristics. Immunology3 cells are characterized by the expression of B cell markers including Cd19, Cd79a, and Cd79b, and Immunology4 cells are marked by macrophage-associated genes such as Lyz2 and Cd68 (Fig. 3B).

To further investigate their relationship with corresponding immune cells and the age-related changes in their cellular and molecular characteristics, we integrated splenic immune cell data from the *Tabula Muris* datasets [28, 31] (B cells, Mast cells, Macrophages) and splenic DCs from a prior study [32]. The feature plot (Fig. 3A) illustrates that the positions of these immunology-related ECs are distinct from those of classical ECs, suggesting functional divergence. We then assessed the correlation between classical ECs, immunology-related ECs, and immune cells using a defined methodology. Classical ECs (Capillary arterial, Capillary venous, Capillary1, Capillary2, Artery, and Proliferating) showed strong inter-correlations with coefficients exceeding 0.8 (Fig. S1 E). In the young cohort, the correlation between classical ECs and immunology-related ECs ranged from 0.5 to 0.8, lower than within the classical group, whereas in the older cohort, the correlation coefficients were higher. The correlation analysis between immunology-related ECs and immune cells (Fig. 3C) revealed that Immunology1 and DC had the strongest association, particularly in the older group. In the young cohort, B cell and Immunology3, as well as Mast cells with Immunology2 and Macrophages with Immunology4, shared stronger relationships.

Then we further analyzed the differential expression genes between immunology-related ECs and their relative immune cells (Fig. S5 A-D). We calculated the DEGs between them and the top5 DEGs are shown. The presence of endothelial cell markers Gm42418 among all the upregulated DEGs in four immunology-related EC clusters indicates their characteristics. Besides, immune cell related genes also presented in the up-regulated DEGs in each immunology-related EC clusters such as Ly6a in Immunology1(Fig. S5 A), Mcpt8 in Immunology2 (Fig. S5 B), Ighm and Igkc in Immunology3(Fig. S5 C) and Adgre1 in Immunology4(Fig. S5 D), illustrating that immunology-related ECs possesses attributes of both endothelial cells and immune cells, representing an intermediate state of endothelial cells while retaining the conservatism of endothelial cells.

To gain a deeper understanding of immunology-related ECs, we compared them with classical ECs using the Immunology-Related Gene Set Score [14] (Fig. 3D, Methods). It is clear that the scores of immunology-related ECs exceed those of classical ECs in both young and old groups, reinforcing our hypothesis that immunology-related ECs possess enhanced immune regulatory functions. When comparing within classical and immunology-related ECs, the scores for the young group are higher than those for the old group, aligning with previous studies that suggest a decline in the immune regulatory functions of ECs with age [23].

Previous research has highlighted the role of ECs in the recruitment and homing of immune cells [33, 34].By interacting with circulating innate and adaptive immune cells and regulating their extravasation from the bloodstream into the tissue parenchyma, ECs could play a crucial role in controlling tissue and lymph node inflammation. Therefore, we calculated the Immune Cell Homing (ICH) score [15] in classical ECs and immunology-related ECs (Fig. 3D). In both young and old groups, classical ECs exhibit higher scores than immunology-related ECs, suggesting that immunology-related ECs have transitioned away from their endothelial characteristics and have adopted properties more akin to immune cells.

Given that ECs are considered semi-professional APCs [10], we evaluated the antigen presentation score between classical ECs and immunology-related ECs to discern any differences (Fig. S1 F). The higher scores of both young and old immunology-related ECs compared to classical groups support the notion that immunology-related ECs may possess stronger immunomodulatory functions than classical ECs.

The findings discussed above led us to consider whether the four identified immunology-related ECs might align with the concept of immune cell-like ECs (EndICLT) [11]. We proceeded to evaluate the unique immunoregulatory functions of these four immunology-related ECs in relation to their associated immune cells. For instance, DCs are renowned for their role as efficient APCs [35], prompting us to score this function among the four immunology-related ECs. The highest antigen presentation score was observed in Immunology1 ECs in both young and old groups (Fig. 3E), suggesting a strong functional resemblance to DCs. This similarity extends beyond shared markers to encompass functional attributes, with aging appearing to enhance antigen presentation capabilities.

For Immunology2, we calculated the allergy gene score, given the central role of Mast cells in initiating allergic immune responses (Fig. 3F) [36]. Immunology1, Immunology2, and Immunology4 scored similarly, reflecting the complex interplay of various immune cells and molecules involved in allergic responses. In terms of B cell activation, Immunology3 demonstrated the strongest performance, underscoring its close association with B cells, and this function was preserved with aging. For Immunology4, we evaluated the phagocytosis score, and as anticipated, it scored the highest among the immunology-related EC groups. The older group achieved lower scores compared to the younger group, aligning with previous studies that suggest the phagocytic function of Macrophages is impaired with age [37].

In order to explore if the age-induced changes in immunology-related ECs are consistent with the immune cell in peripheral blood. We used the peripheral blood scRNA-seq data in the young and aged mouse from article “Single-cell transcriptomics of peripheral blood in the aging mouse”(GSE120505) [38]. After quality control, we reclustered and identified 10 clusters of cells in human peripheral blood cells (Fig. S6 A). The subcluster related to immunology-related EC (DC, Basophil, B cell and Macrophage) was extracted and aged-related DEGs of each cluster were analyzed (Fig. S6 B-E).

In peripheral blood, age-related changes in DCs are linked to blood coagulation, while in DC-resembling ECs (Immunology1), they are associated with antigen processing and extracellular matrix organization. Three intersecting genes were found among the upregulated DEGs in peripheral blood DCs and Immunology1(Fig. S6 B). One of them is Ecm1, upregulated in the aging heart, contributes to cardiac fibroblast stimulation and fibrosis in aging and myocardial infarction [39]. In peripheral basophils, upregulated DEGs are linked to leukocyte chemotaxis, while in mast cell-resembling ECs (Immunology2), they are related to vasculature development. There were no overlapping genes between the age-upregulated DEGs in basophils and Immunology2 (Fig. S6 C), indicating heterogeneous changes during aging despite their shared origin. For peripheral macrophages, the function of regulating response to external stimuli was upregulated. In macrophage-resembling ECs (Immunology4), age-related DEGs were associated with blood vessel endothelial cell migration. Psen1, relevant to autosomal-dominant Alzheimer’s disease, was among the overlapping genes (Fig. S6 E) [40].

These results provide evidence that peripheral blood immune cells and immunology-related endothelial cells share some similar age-induced changes in both functional and gene phenotypes such as immune response and chemotaxis. However, the aging-related changes in clusters of immunology-related endothelial cells are more focused on their regulatory function in vessel development.

Overall, Immunology-related ECs are not only express markers similar to immune cells but also retain their immunomodulatory functions. This further substantiates the notion that they are undergoing EndICLT.

### Age-related molecular changes along differentiation trajectories

**Fig. 4:**
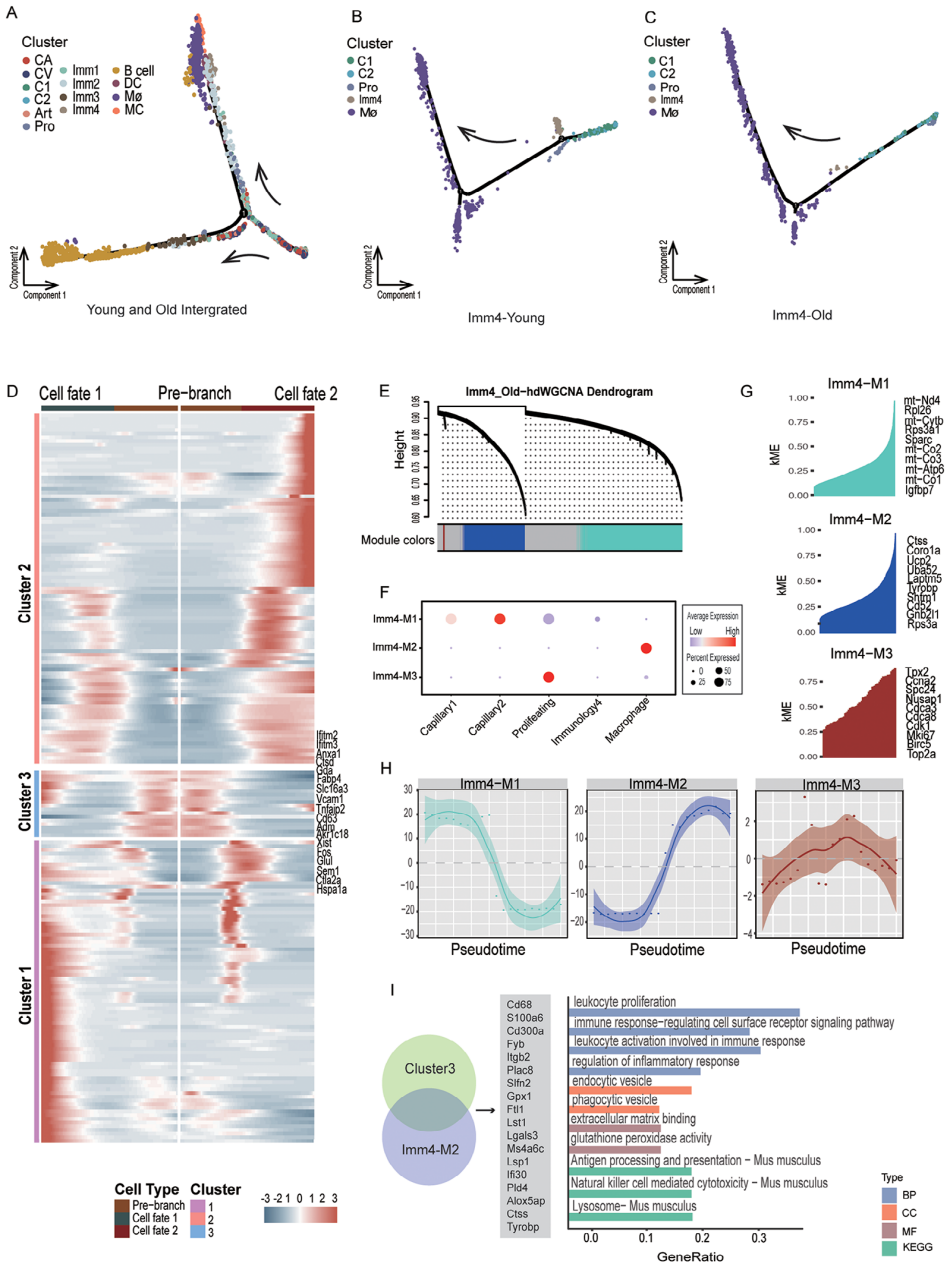
The pseudotime trajectory reveals relationship between splenic immunology-related ECs and immune cells. **(A)** Pseudotime analysis conducted on all ten ECs cell types (Capillary arterial, Capillaryvenous, Capillary1, Capillary2, Artery, Immunology1, Immunology2, Immunology3, Immunology4, Prolifearting) in splenic ECs (young and old integrated) and 4 groups of Immune cells (DC, Mast cells, B cells and Macrophages). The points are colored by celltypes. **(B)** Pseudotime trajectory analysis of Immunology4 EC related subtypes in young group: Capillary1, Capillary2, Proliferating, Immunology4 and Macrophages. **(C)** Pseudotime trajectory analysis of Immunology4 EC related subtypes in old group: Capillary1, Capillary2, Proliferating, Immunology4 and Macrophages. **(D)** Heatmap showing the expression profiles along the pseudotime of Time differential genes (q value < 1e-4) in the trajectory of Immunolog4 EC related subtypes in old group including Capillary1, Capillary2, Proliferating, Immunology4, and Macrophage, which were divided into three clusters with the expression pattern and the gene in cluster 3 represented on the right. **(E)** The cluster dendrogram of co-expression in the trajectory of Immunology4 EC related subtypes in old group. **(F)** Dotplot showing the expression of the co-expression modules in Immunology4 EC related subtypes in old group. **(G)** The kME plot of the co-expression modules in Immunology 4 EC related subtypes. Top 10 hub genes of each co-expression module of Immunology4 EC related subtypes in old group are visualized on the right. **(H)** The pseudotime trajectory for co-expression module. The Y-axis represents the kME score of each hub genes. **(I)** Left, pie plot showing overlapped genes between cluster 3 and hub genes of co-expression module 2 of the Immunology 4 EC related subtypes. Right, bar plot showing enriched GO terms and KEGG of the overlapped genes listed in the middle

Previous research suggests that cells undergoing EndICLT do not fully differentiate into immune cells but rather occupy an intermediate state between ECs and immune cells [11]. To investigate whether the four immunology-related ECs fall along differentiation trajectories, we conducted pseudotime analysis using Monocle 2 [41, 42]. We first analyzed all splenic ECs and immune cells in both young and old groups (Fig. 4A). The analysis delineated three major differentiated cell groups: (1) ECs; (2) B cells; (3) Macrophages and DCs. It appeared that the four immunology-related EC groups were positioned along these trajectory paths.

To gain a clearer understanding of the status of these four groups, we divided the analysis into four subsets (Fig. S2 A, B). The first subset included Capillary1, Capillary2, Proliferating, Immunology1, and DCs, with Proliferating ECs as the starting point due to their regenerative potential (Fig. S2 A-i). Some Immunology1 cells were located on the branch between ECs and DCs, in State 3, suggesting a potential transitional status (Fig. S2 B-I). The second subset comprised Capillary1, Capillary2, Proliferating, Immunology2, and Mast cells. In the older group, Proliferating and Immunology2 cells branched towards the Mast cells trajectory, suggesting that aging may disrupt EC differentiation (Fig. S2 A-ii, Fig. S2 B-ii). Immunology2 cells also occupied a middle status between ECs and Mast cells.

The third subset included Capillary1, Capillary2, Proliferating, Immunology3, and B cells (Fig. S2 A-iii, Fig. S2 B-iii), with all Immunology3 cells positioned in an intermediate state between ECs and immune cells. The fourth subset contained Capillary1, Capillary2, Proliferating, Immunology4, and Macrophages. Similar to the third subset, all Immunology4 ECs were situated in an intermediate state (Fig. S2 A-iv, Fig. S2 B-iv). Notably, the proximity of Immunology4 ECs to the branch point of the trajectory prompted us to consider potential molecular changes in Immunology4 that could facilitate the transition from classical ECs to Macrophages.

To further explore the age-related changes, we separated the Immunology4-related ECs into young and old groups and conducted individual trajectory analyses. The trajectory analysis revealed distinct patterns between the young and old groups (Fig. 4B, C). We used the cellAlign algorithm [21] to analyze the distinctions between the trajectories of old and young. First, we analyzed the global alignment to quantify overall similarity in expression throughout the trajectory (Fig. S7 A, B). Then we used the young dataset as the reference to map the difference between the two datasets(Fig. S7 C).The results indicated that during pseudotime, most of the genes in young and old groups are conserved while some of the genes are unique. Secondly, we analyzed the local alignment to identifies regions of the trajectory that are closer to each other (Fig. S7 D). The regions are remained conservative within the 0-100 range while in the later stages of the trajectory, they begun to differentiate. This clearly demonstrates the differences in trajectories between young and old group, highlighting specific gene changes in the differentiation trajectory associated with aging.

Within the older group, Immunology4 ECs were found to occupy an intermediate state between ECs and immune cells, a pattern not observed in the young group (Fig. 4C). This observation suggests that aging may push Immunology4 into a transitional state.

To substantiate our hypothesis, we initially examined the gene expression profiles at the branch point in the aged group of Immunology4 ECs related subsets (Fig. 4D). The genes were classified into three groups according to their expression dynamics. It was clear that the majority of genes in Cluster 1 displayed high expression levels in Cell Fate 2, while those in Cluster 2 were predominantly expressed in Cell Fate 2 as well. In contrast, Cluster 3 exhibited high expression levels before the branch point.

Subsequently, we identified co-expression modules using the R package hdWCGNA [17] to verify whether gene expression alters or remains consistent throughout the transition process from ECs to immune cells in the aged group. We constructed a co-expression network and visualized it using a dendrogram (Fig. 4E), and three co-expression modules were calculated. Genes within Module 1 showed elevated expression levels in Proliferating ECs and Capillary1 and Capillary2 ECs, while those in Module 2 and 3 displayed heightened expression in Macrophages (Fig. 4F).

We then computed the Module Eigengenes in each module, a metric frequently used to summarize the gene expression profile of an entire co-expression module. The module eigengenes were computed by performing principal component analysis (PCA) on the subset of the gene expression matrix comprising each module [17]. The top 10 hub genes of Module 1 (Fig. 4G-upper) are primarily involved in the regulation of endothelial cell proliferation and regulation of vascular permeability. For Module 2, the hub genes mainly focus on the regulation of lymphocyte proliferation and positive regulation of leukocyte-mediated cytotoxicity (Fig. 4F-middle). Module 3 hub genes are related to the positive regulation of the mitotic cell cycle phase transition. We analyzed how the module eigengenes change throughout the pseudotime trajectories for each co-expression module using the hdWGCNA function PlotModuleTrajectory.

We conducted pseudotime trajectory analysis with Monocle2 and studied module dynamics throughout the cellular transitions from Proliferating ECs to Macrophages. The trajectories indicated that Module 1 was turning off their expression programs throughout the transition from ECs to Macrophages, while Module 2 was turning on in the process. These results paralleled the observation that its endothelial function declined during the transition while its immune phenotype was upregulated. The Module 3 genes remained static during the transition (Fig. 4H). This finding suggested that the genes in Module 2 may contribute to the regulation of the transition.

To identify potential transitional genes, we overlapped genes in Cluster 3 and Module 4’s top 50 hub genes, and 18 genes were detected (Fig. 4I). The GO enrichment showed the function of overlapped genes mainly about regulation of inflammatory response (Gpx1, Alox5ap, Ctss), extracellular matrix binding (Ctss, Lgals3), antigen processing and presentation (Ifi30, Ctss), and phagocytic and endocytic vesicle (Ctss, Pld4, Ftl1). Previous research has shown that Ctss is involved in inducing the release of inflammatory cytokines and leading to endothelial dysfunction in hyperglycemic conditions, and another study demonstrated that decreasing Ctss can ameliorate age-related dry eye. These findings suggested that aging may activate Ctss and initiate the EndICLT process (Fig. S2 C).

Besides, we also analyzed the transcription factors in the old Immunology4 subsets (including Capillary1, Capillary2, Proliferating, Immunology4 and Macrophage) used pySCENIC [19]. After constructing the co-expression modules, we calculated the cell-type specific regulators(Fig. S8 A, B).Then to explore the TFs changes in trajectory, we integrate transcription factor activity into the pseudotime matrix(the pseudotime was normalized). According to our pervious trajectory (Fig. 4C), the transition occurred in the time interval between 0.35 and 0.45 (from 0 to 1). We specifically investigated immunology4-specific transcription factors and plotted the transcription factor activity over pseudotime to see whether any specific changes near transition time(Fig. S8 C).We found that Ltf showed a specific upregulation around 0.4(Fig. S8 D). It is well-known that LTF could activate the NF-κB signaling pathway, promoting macrophage activation [43]. For example, LTF binding to CD14 receptor competes with the bacterial LPS (product of dying bacteria) [44] and can attenuate NF-κB-induced transcription of genes for various inflammatory mediators [45]. Also, it is reported that high LTF expression might contribute to meniscal aging and degeneration through the NF-κB signaling pathway [46].Therefore, we could speculate that the endothelial-immunology transition is regulated by Ltf.

### Profiling the impact of aging on spleen ECs cellular communication and molecular signaling pathways

**Fig. 5:**
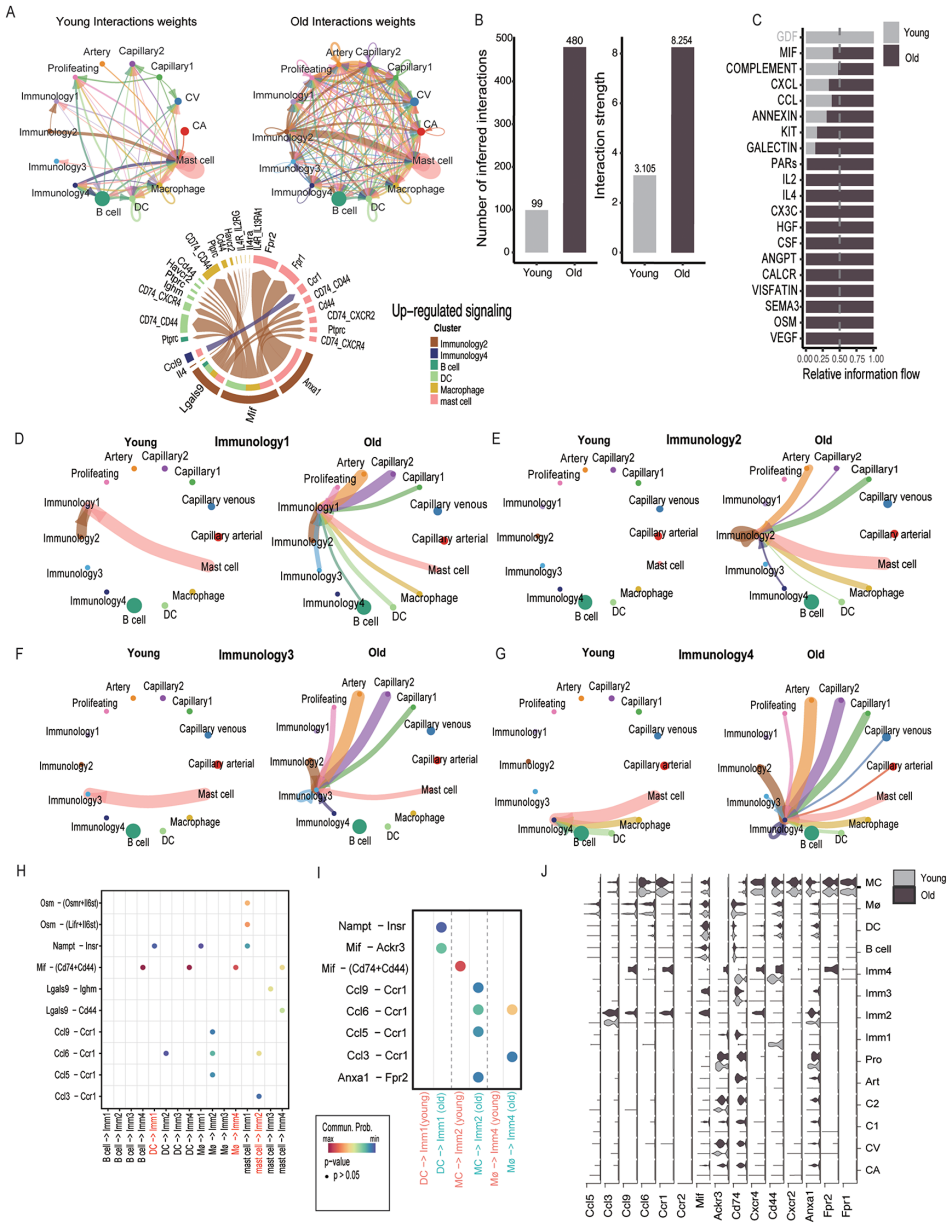
The young and old splenic endothelial-immune cells interactome predictions. **(A)** Circle network diagram of cell-cell interaction patterns of different celltypes in young (left) and old (right) splenic ECs and immune cells, arrows and edge color indicate direction(ligand-receptor), and the edge thickness indicates the sum of the interaction number between populations. Bottom: Chord chart showing the up-regulated signaling pathway between young and old splenic ECs and immune cells. **(B)** Histogram counts the number and interaction strength of cell-cell interactions in young (grey) and old (purple taupe) splenic ECs and immune cells. **(C)** Bar graph shows predicted pathways for young and old splenic ECs and immune cells, with gray labels representing pathways in the young group, black representing pathways shared by the young and old groups, and the purple taupe color representing pathways in the old group. **(D)** Circle network diagram showing the cell-cell interaction patterns in young (left) and old (right) splenic ECs and immune cells targeting on Immunology1 EC. **(E)** Circle network diagram showing the cell-cell interaction patterns in young (left) and old (right) splenic ECs and immune cells targeting on Immunology2 EC. **(F)** Circle network diagram showing the cell-cell interaction patterns in young (left) and old (right) splenic ECs and immune cells targeting on Immunology3 EC. **(G)** Circle network diagram showing the cell-cell interaction patterns in young (left) and old (right) splenic ECs and immune cells targeting on Immunology4 EC. **(H)** Dotplot representing the ligand-receptor pair between splenic ECs and immune cells (young and old intergrated), which splenic ECs are set as target. The specific interaction between splenic EC and immune cells were labeled in red. **(I)** Dotplot representing the ligand-receptor pair of specific interaction between young (coral) and old(cyan) splenic ECs and immune cells (DC-Imm1; MC-Imm2; Macrophage-Imm4). **(J)** Violin plot showing the gene expression of the indicated pathways between splenic ECs and immune cells

To explore cell-to-cell interactions between the young and old groups, we utilized the R package CellChat for analysis [20]. In line with observations from other studies [23, 47], the aged group exhibited stronger signal communication in terms of both quantity and intensity (Fig. 5A, B). Compared to the young group, inflammation pathways such as Ccl, Cxcl, and Mif were upregulated (Fig. 5A-lower, C). Pathways in the old Immunology2 ECs were the most upregulated (Fig. 5A-lower).

Previous research has confirmed the interaction between tumor-enriched angiogenic ECs and Tumor-associated Macrophages (TAMs) through TGFB1 (angiogenic ECs) ⇔ TGFBR1 (Mye1, TAMs) [12]. However, the potential mechanisms by which ECs can modulate immune cells during aging are still under investigation. We focused our analysis on the EC-immune cell interactome by comparing the signals of four immunology-related ECs between the young and old groups(Fig. 5D-G). We first designated the four immunology-related ECs as targets to examine how immune cells and other endothelial cells interact with them. Compared to the young group, Immunology1, Immunology 2, and Immunology4 all received signals from their related immune cells (Fig. 5D, E, G). We then further analyzed the interaction between the four immunology-related ECs and their related immune cells (Fig. 5H).

In the aging group, DCs interacted with Immunology1 through Nampt ⇔ Insr and Mif ⇔ Ackr3 (Fig. 5I). Ackr3 has been shown to be involved in the trans-endothelial migration of immune cells, enabling arterial invasion and accumulation of immune cells in lesions, causing atherosclerosis, and is upregulated with aging (Fig. 5J) [48, 49]. Mast cells interacted with Immunology2 through Ccl5⇔Ccr1, a specific pathway that recruits monocytes into inflamed tissues by primarily triggering Ccr1-mediated arrest on endothelial cells, and has been shown to promote hematogenous metastasis in colorectal cancer [50, 51]. The interaction between aging Macrophages and Immunology4 is primarily through Ccl3⇔Ccr1 and Ccl6⇔Ccr1. Ccr1 plays a significant role in the bone microenvironment, and Ccl3 is considered an osteoclast activating factor produced by MM plasma cells, further stimulating osteoclastogenesis [52]. Other research has shown that Ccl3 and Ccr1 expressed by tumor-associated Macrophages is associated with enhanced interaction with breast carcinoma cells and metastatic seeding to the lung [53]. Ccl6 is considered as a chemotactic agent for monocytes and Macrophages and is produced in exaggerated quantities in inflammatory and remodeling disorders [54].

We then designated the four immunology-related ECs as senders to examine how they interact with immune cells (Fig. S3 A-D-G). Compared to the young group, aging Immunology1 ECs exert unique signaling effects on DCs (Fig. S3 E) through Kitl ⇔ Kit. Endothelial cells have been shown to maintain Hematopoietic Stem Cells at homeostasis by expressing Kitl [55, 56]. For the interaction of Immunology2 and Mast cells, the Anxa1 ⇔ Fpr2/ Fpr1 signal was upregulated. Anxa1 has been shown to trigger angiogenesis such as cell functional migration and invasion in Pancreatic cancer. This validation confirmed that Anxa1 not only plays a role in EndMT [57] but also exerts regulatory control over EMT in papillary thyroid carcinoma [58]. We can speculate on the potential role of Anxa1 in regulating EndICLT during the aging process.

In summary, these cellular interaction findings indicate an upregulation of inflammatory activation during spleen aging, and the enhanced cell-cell interactions may lead to the reprogramming of ECs.

### Further validation of the existence of immunology-related ECs in other mouse organs and humans

To validate whether the presence of immunology-related ECs is spleen-specific characteristic, we analyzed scRNA-seq in other mouse organ(heart, liver and kidney) from our previous studies [23, 59, 60]. We integrated three datasets and identified two immunology-related clusters (Fig. S9 A). Most of the immunology-related ECs are located in the liver (Fig. S9 C), consistent with our previous analysis of liver endothelial cells [59]. Additionally, these immunology-related ECs are predominantly found in aged mice. Then we analyzed the top 10 marker genes in those two immunology-related clusters (Fig. S9 B) and found there are similar markers between splenic ECs and EC_merge immunology-related clusters such as Cd52, Lyz2. We further compared the correlation between two EC_merge immunology-related clusters and four splenic EC clusters (Fig. S9 D, E). EC_merge immunology1 is most similar to Immunology4 (Correlation coefficient:0.71) and EC_merge immunology 2 is most similar to Immunology1 (Correlation coefficient:0.72). These results indicate that while immunology-related endothelial cells are present in other mouse organs, the splenic immunology ECs exhibit greater diversity compared to those in other organs.

These findings prompted us to investigate the presence of immunology-related ECs in the human spleen. We analyzed human splenic endothelial cells from the *Tabula Sapiens* dataset [61]. After quality control and removal of doublets, we reclustered the endothelial cells and identified 7 clusters (Fig. S10 A, B). First, we identified the presence of homologous genes for immune-related EC marker genes in the human clusters, including H2-Eb1—HLA-DRB1/HL1-DRB5, Fcer1a—FCER1A, CD79a—CD79A, and Lyz2—LYZ (Fig. S10 C). The homologous genes for the Immunology4 marker (LYZ) were expressed in cluster 7 of the human subset. We further confirmed the specific expression of macrophage markers CD163 and CD68, which exhibited distinctive patterns (Fig. S10 C). Subsequently, we analyzed the top 30 marker genes of the human immunology-related ECs and their GO functions (Fig. S10 D). Similar to our findings in mouse splenic ECs (especially Immunology4), the GO enrichment was highly related to immune response and phagocytosis.

Overall, these results provide evidence for the existence of immune-related endothelial cells in various mouse organs and the human spleen.

## Discussion

The exploration of aging’s influence on splenic EC heterogeneity remains underrepresented in scientific literature, despite the critical role these cells play in orchestrating immune responses [62].The spleen’s high vascularity positions its ECs as pivotal facilitators of interactions between lymphocytes and antigen-presenting cells, essential for robust immunological defense. Recent studies have underscored the transcriptional diversity of splenic ECs and their involvement in key functions such as scavenging and lipid metabolism, which are vital for maintaining splenic homeostasis [22]. However, the regulatory effects of aging on these cells and their interactions with immune cells have not been thoroughly investigated. To address this gap, we have endeavored to construct a comprehensive atlas and analyze the aging-related effects and mechanisms on various vascular ECs.

We have successfully generated the inaugural single-cell atlas of aged splenic ECs, delineating 10 distinct EC subtypes (Fig. 1B) after quality control and doublets checking (Fig. S4). Beyond the previously recognized cell types [22], our study has unveiled novel subpopulations, including four immunology-related EC types (Immunology1-4) and a subset of Proliferating ECs. GO analysis validated the biological functions of these ECs, with the immunology-related ECs notably expressing markers typically associated with immune cells (Table S1).We further investigated the overlap of these markers between the immunology-related ECs and immune cells (Fig. 3B), with GO analysis corroborating the functional attributes of these ECs.

Our analysis then focused on elucidating common regulatory mechanisms of aging within splenic ECs. We observed that the most significant changes in differentially expressed genes (DEGs), both upregulated and downregulated, were predominantly found in Immunology1, Immunology4, and Proliferating ECs (Fig. S1 C). This suggests that these cell types are particularly susceptible to the effects of aging. The aged endothelial microenvironment is characterized by heightened inflammation, as evidenced by the expression of immunology gene signatures such as H2-Aa, H2-Ab1, H2-Eb1, and Cd74 (Fig. 3D). The upregulation of functions related to the “inflammatory cellular response to interferon-beta” and “antigen processing and presentation of exogenous peptide antigen via MHC class II” aligns with the hypothesis that interferon activation is a concurrent process in spleen aging, supporting theories from prior studies [63]. Our findings are consistent with those reported in the literature [23, 24, 64], and we have further illuminated the heterogeneity (Fig. 2D) and the aging-related effects on various cell subpopulations (Fig. S1 C, D) using diverse analytical approaches.

Significantly, we have characterized four groups of immunology-related ECs - Immunology1, Immunology2, Immunology3, and Immunology4 - as EndICLTs. These cells are in a transitional state, not yet fully differentiated into immune cells. This concept aligns with findings from studies on carotid artery endothelial cells under disturbed flow (d-flow), which suggested a role for such cells in the progression of atherosclerosis [11]. In these studies, the E8 cell type was classified as an EndICLT due to its high expression of several macrophage gene markers. In our research, we expanded this concept, associating Immunology1 with DCs, Immunology2 with Mast cells, Immunology3 with B cells, and Immunology4 with Macrophages. This association was based on the overlap of marker genes between the four immunology-related ECs and these immune cells (Fig. 3B), with these markers being highly expressed in their respective subtypes. The differential expression genes between immunology-related ECs and their relative immune cells (Fig. S5 A-D) showed that the presence of endothelial cell markers Gm42418 among all the upregulated DEGs in four immunology-related EC clusters. Besides, immune cell related genes also presented in the up-regulated DEGs in each immunology-related EC clusters such as Ly6a in Immunology1(Fig. S5 A), Mcpt8 in Immunology2 (Fig. S5 B), Ighm and Igkc in Immunology3(Fig. S5 C) and Adgre1 in Immunology4(Fig. S5 D), illustrating that immunology-related ECs possesses attributes of both endothelial cells and immune cells, representing an intermediate state of endothelial cells while retaining the conservatism of endothelial cells.

To validate our hypothesis, we conducted GO analysis and correlation tests, which confirmed the similarity between these cell types (Fig. 3A, C). We also compared the immunology-related ECs with classical ECs using gene set score analysis (Fig. 3D). This comparison revealed that, relative to classical ECs, the four immunology-related ECs possess enhanced immune-regulating capabilities and are losing their endothelial cell phenotypes. We further explored the specific functions of the four immunology-related clusters. For Immunology1, we assessed the antigen-presenting score (Fig. 3E). Given that endothelial cells are considered semi-professional APCs [10], we first compared classical ECs and immunology-related ECs to eliminate interference (Fig. S1 F). Our analysis confirmed that immunology-related ECs have higher APC function, with Immunology1 scoring the highest, thereby supporting its association with DCs (Fig. 3F). In Immunology2, we evaluated the allergy score, with Immunology1, Immunology2, and Immunology4 exhibiting comparable scores, reflecting the complex interplay of different immune cells and molecules in allergic responses. Immunology3 scored highest in B cell activation, indicating its close association with B cells. For Immunology4, we assessed the phagocytosis score, and as anticipated, it scored the highest among the immunology-related EC group. The analysis of the peripheral blood scRNA-seq data in the young and aged mouse [38](Fig. S6 A) showing that in peripheral blood, age-related changes in DCs are associated with blood coagulation, whereas in Immunology1, they relate to antigen processing and extracellular matrix organization. One of the intersect genes, contributes to cardiac fibroblast stimulation and fibrosis in aging [39]. For peripheral macrophages, the function of regulating response to external stimuli was upregulated while in Immunology4, age-related DEGs were associated with blood vessel endothelial cell migration. Psen1, relevant to autosomal-dominant Alzheimer’s disease [40], was among the overlapping genes(Fig. S6 E). These provide evidence that peripheral blood immune cells and immunology-related endothelial cells share some similar age-induced changes in both functional and gene phenotypes such as immune response and chemotaxis. However, the aging-related changes in clusters of immunology-related endothelial cells are more focused on their regulatory function in vessel development.

Chronic d-flow has been established to induce the EndICLT phenotype [11]. This led us to question whether aging could similarly trigger EndICLT. To probe this possibility, we performed pseudotime trajectory analysis on selected EC subtypes (Fig. 4A, B). Our analysis revealed that among aging Immunology4-related ECs (Proliferating, Capillary1, Capillary2, Immunology4, and Macrophages), the Immunology4 subtype was predominantly transitioning towards immune cell-like phenotypes (Fig. 4C). The young and old Immunology4 subtype was proven showing a deviate in the later stages (Fig. S7 D) highlighting specific gene changes in the differentiation trajectory associated with aging.

The EndICLT hypothesis was further substantiated by conducting gene consensus co-expression network analysis (Fig. 4E-G) and trajectory branch heatmap visualization (Fig. 4D). By overlapping the highly expressed genes before the pre-branch and the co-expression module genes upregulated throughout the trajectory, we identified Ctss as a potential regulator of the EndICLT process in aging. This hypothesis aligns with previous reports, which have shown that Ctss is highly upregulated in immune cells, particularly microglia, in the aging mouse brain [65] and retina [66]. Furthermore, Ctss has been implicated in the inflammatory processes of immune diseases such as atopic dermatitis, psoriasis, bronchial asthma, and rheumatoid arthritis [67, 68], as well as age-related dry eye [69].

Notably, Ctss knockdown significantly reduced the expression of transcription factors nuclear factor-kappa B (NF-κB) and inducible NO synthase (iNOS), along with vascular endothelial growth factor A (VEGFA), intercellular adhesion molecule 1 (ICAM-1), and vascular cell adhesion molecule 1 (VCAM-1) [70]. Given that NF-κB is a key inducer of endothelial fibrosis in EndMT and ICAM-1 and VCAM-1 are markers of EndMT [71], these findings suggest that Ctss may be a regulatory gene of EndICLT in the aging mouse spleen. However, further lineage tracing studies are needed to solidify the EndICLT hypothesis.

We also analyzed the transcription factors changes in pseudotime trajectory in the old immunology4 subsets. According to our pervious trajectory (Fig. 4C), the transition occurred in the time interval between 0.35 and 0.45 (from 0 to 1). We specifically investigated immunology4-specific transcription factors(Fig. S8 A, B) and plotted the transcription factor activity over pseudotime(Fig. S8 C).We found that Ltf showed a specific upregulation around 0.4(Fig. S8 D). It is well-known that LTF could activate the NF-κB signaling pathway, promoting macrophage activation [43].Also, it is reported that high LTF expression might contribute to meniscal aging and degeneration through the NF-κB signaling pathway [46].Therefore, we could speculate that the endothelial-immunology transition is regulated by Ltf.

Our findings on EndICLT are supported by previous reports showing that vascular ECs and lymphatic ECs can serve as APCs, expressing major histocompatibility complex class I (MHC class I) and MHC class II [72, 73]. The plasticity of ECs is further evidenced by their ability to transition into osteoblasts and hematopoietic cells [74, 75]. An epithelial cell to immune cell-like transition has also been reported [76].

Also, to further analyze the existence of the immunology-related ECs in other mouse organs and in human splenic ECs. We analyzed scRNA-seq in other mouse organ(heart, liver and kidney) from our previous studies(Fig. S9 A) [23, 59, 60] and found there are similar markers between splenic EC and EC_merge immunology-related clusters such as Cd52, Lyz2. The correlation between this two EC_merge immunology-related clusters and four splenic EC clusters (Fig.S9 C, D) showed that EC_immunology1 is most similar to Immunology4 (Correlation coefficient:0.71) and EC_immunology 2 is most similar to Immunology1 (Correlation coefficient:0.72). These results indicate that while immunology-related endothelial cells are present in other mouse organs, the splenic immunology ECs exhibit greater diversity compared to those in other organs.

We analyzed human splenic endothelial cell from dataset *Tabula Sapiens* [61] (Fig. S10 A). The marker of Immunology4 (LYZ) was expressed in the cluster7 in human subset. Further we validated the expression of macrophage markers (CD163,CD68) and both showed a specific expression (Fig.S10 C). the GO enrichment highly related to immune response and phagocytosis (Fig. S10 D). All these findings provide evidence about the existence of immune-related endothelial cells in human splenic endothelial cells.

In line with other reports, we found that aging weakens many immune-regulating abilities to varying degrees. Aging has been shown to significantly impact the physiological function of immune-related cells in the spleen, impairing both humoral and cellular immunity as indicated by increased apoptosis and reduced numbers of B cells and T cells in the spleen [7]. There is a confirmed decrease in the abundance of naive CD8 + T cells in the spleen, and new subpopulations have been identified in age-associated immune cells in the spleen, including cytotoxic CD4 T cells and activated regulatory T cells (aTreg) [7, 77]. Additionally, age-dependent deficiencies in the functional immunity of the spleen may be due to the reorganization of splenic microanatomy, such as the boundary between T cells and B cells becoming less defined in older mice and a loss of marginal zone Macrophages [5, 6].

Finally, we examined age-related alterations in intercellular communication between endothelial cells and immune cells, with a particular emphasis on the interaction between immunology-related ECs and immune cells. Our findings revealed that aging immunology ECs engage in unique signaling pathways with their immune cell counterparts. We also identified several signaling pathways that may contribute to the reprogramming of endothelial cells during aging.

Despite these findings, our study has several limitations. Firstly, we acknowledge the need for future protein analysis and functional validation experiments to confirm the role of each EC phenotype. Secondly, the hypothesis of EndICLT in aging requires further substantiation through in vitro results. Lastly, we cannot rule out the possibility that some EC subtypes may have been lost during tissue dissociation and MACS-based EC enrichment.

## Conclusion

In summary, by constructing this single-cell resolution transcriptome atlas of the splenic endothelium, we have gained a deeper understanding of age-related changes in splenic endothelial cells. Through this atlas, we not only pinpointed the molecular characteristics linked to increased inflammation and interferon activation function in aged spleens but also uncovered potential transitional states of splenic endothelial cells and their interactions with immune cells during the aging process. We believe this study offers valuable insights into the heterogeneity and reprogramming of splenic ECs in aging, as well as their role in immunosenescence.

### Electronic supplementary material

Below is the link to the electronic supplementary material.

Supplementary Material 1

Supplementary Material 2

Supplementary Material 3

Supplementary Material 4

## Data Availability

The scRNA-seq data is stored in NGDC database (National Genomics Data Center) (HRA004465).

## Abbreviations

ECs: Endothelial cells
UMAP: Uniform Manifold Approximation and Projection
DEGs: Differentially Expressed Genes
GO: Gene Ontology
EndICLT: Endothelial-to-Immune-like Cell Transition
Ctss: Cathepsin S
EndMT: Endothelial-to-Mesenchymal Transition
EMT: Epithelial-to-Mesenchymal Transition
d-flow: Disturbed Flow
NF-κB: Nuclear factor-kappa B
iNOS: inducible NO synthase
VEGFA: Vascular endothelial growth factor A
ICAM-1: Intercellular adhesion molecule 1
VCAM-1: Vascular cell adhesion molecule 1
