# Supplementary material for: Deciphering the impact of aging on splenic endothelial cell heterogeneity and immunosenescence through single-cell RNA sequencing analysis
Source: Immun Ageing. 2024 Jul 18;21:48. doi: 10.1186/s12979-024-00452-1 (PMC11256597; doi:10.1186/s12979-024-00452-1)
Supplement: Supplementary file 1 — Supplementary Material 1 [file 12979_2024_452_MOESM1_ESM.docx]

**Additional files**

**Additional file 1**：Figure S1


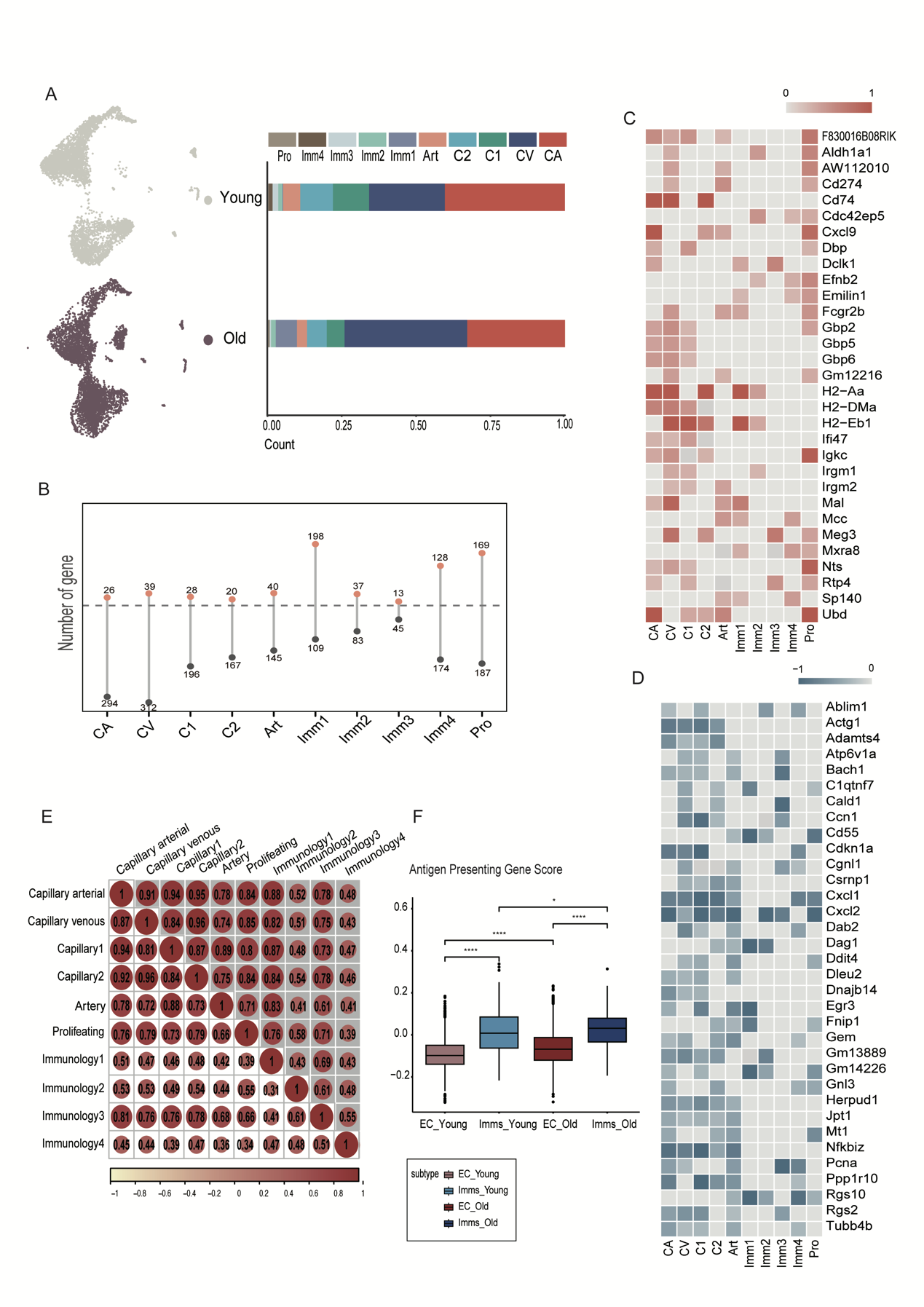


**Figure S1:Comparison between young and aged splenic ECs in different dimensions**

1. Left: Distribution of ECs in the UMAP plots of young and old mouse. Right: The rate of clusters in young and old splenic ECs.
2. Lollipop plot showing the up- and down- regulated DEGs between young and old ECs. The number of DEGs are labeled.
3. Heatmap showing upregulated DEGs in at least 3 cell types in mouse splenic ECs. Only genes with same direction of differential expression among different cell types are included.
4. Heatmap showing downregulated DEGs in at least 4 cell types in mouse splenic ECs. Only genes with same direction of differential expression among different cell types are included.
5. Correlation matrix displaying the relationships between splenic ECs and Immune cells. The upper matrix, shaded in grey, illustrates the correlations between Old splenic ECs and Immune cells, while the lower matrix depicts the correlations for young ones. Each correlation coefficient is labeled within the corresponding circle in the matrix.
6. Boxplot showing the Antigen Presenting gene score of classic ECs and immunology-related ECs. ****p < 0.001.

**Additional file 2**：Figure S2


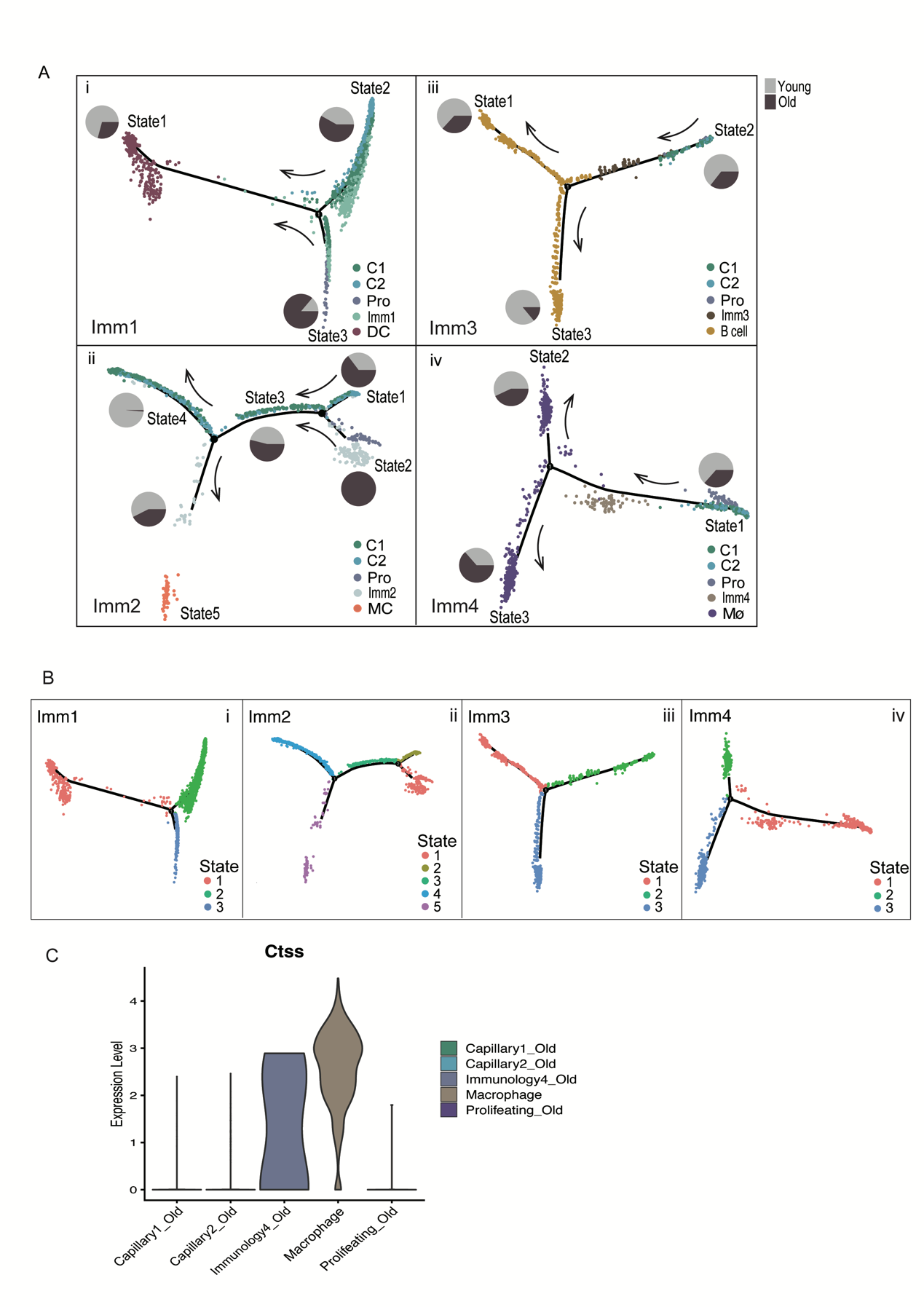


**Figure S2:Pseudotime trajectory between splenic immunology-related ECs and immune cells**

(A) Pseudotime trajectory of specific subtype in splenic ECs and Immune cells. (young and old integrated).

(A-i) Pseudotime trajectory analysis of Immunology1 EC related subtypes: Capillary1, Capillary2, Proliferating, Immunology1 and DCs.

(A-ii) Pseudotime trajectory analysis of Immunology2 EC related subtypes: Capillary1, Capillary2, Proliferating, Immunology2 and Mast cells.

(A-iii) Pseudotime trajectory analysis of Immunology3 EC related subtypes: Capillary1, Capillary2, Proliferating, Immunology3 and B cells.

(A-iv) Pseudotime trajectory analysis of Immunology4 EC related subtypes: Capillary1, Capillary2, Proliferating, Immunology4 and Macrophages.

(B)The Pseudotime analysis showing the states of Immunology ECs related subtypes.

(B-i)The Pseudotime analysis showing the states of Immunology1 EC related subtypes.

(B-ii)The Pseudotime analysis showing the states of Immunology2 EC related subtypes.

(B-iii)The Pseudotime analysis showing the states of Immunology3 EC related subtypes.

(B-iv)The Pseudotime analysis showing the states of Immunology4 EC related subtypes.

(C) Violin plot showing the gene expression of Ctss in old Immunology4 EC related subtypes: Capillary1, Capillary2, Proliferating, Immunology4 and Macrophage.

**Additional file 3**：Figure S3


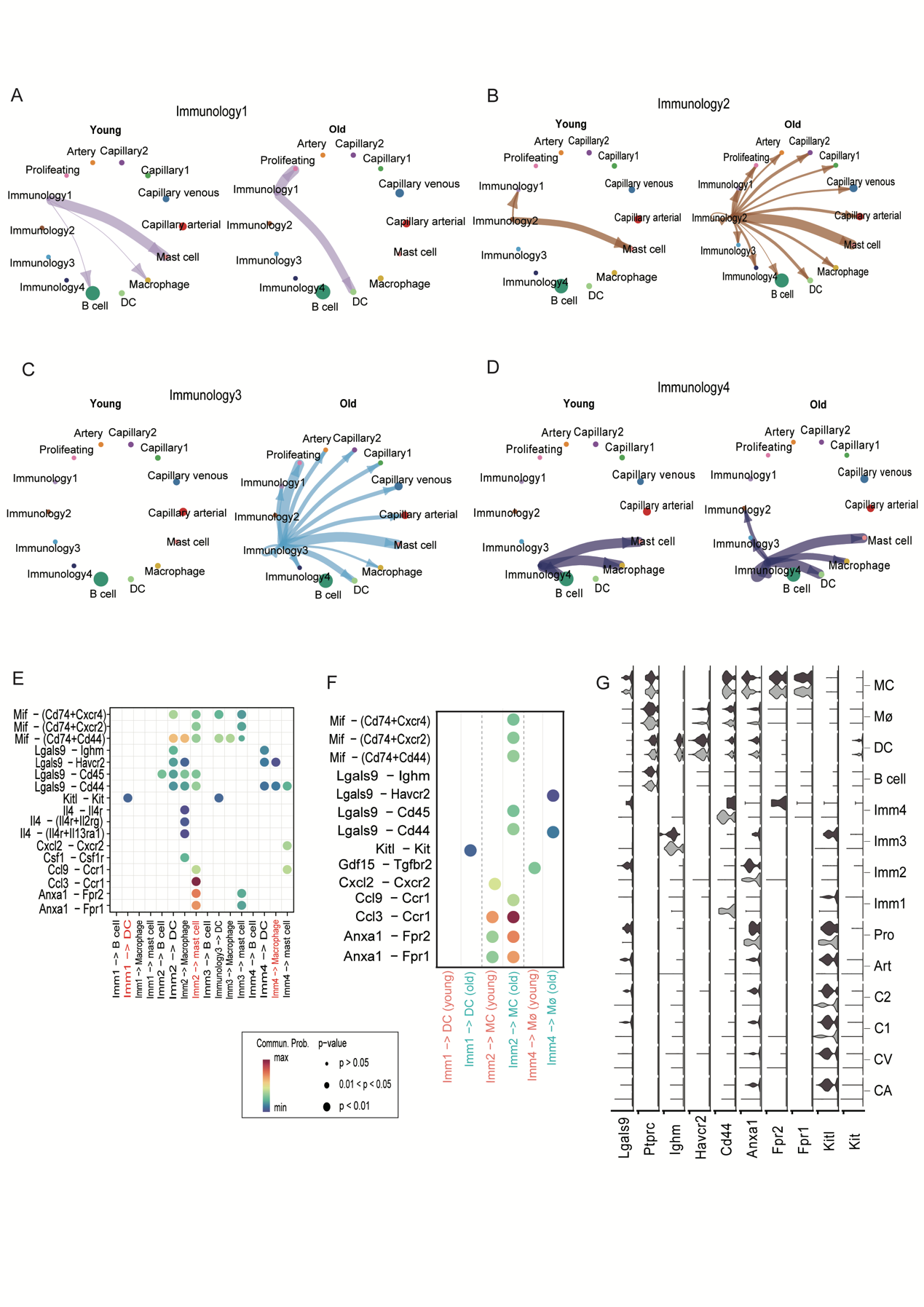


**Figure S3:Cell-cell interaction patterns in young and old splenic ECs and immune cells.**

(A) Circle network diagram showing the cell-cell interaction patterns in young (left) and old (right) splenic ECs and immune cells which sent from Immunology1 EC.

(B) Circle network diagram showing the cell-cell interaction patterns in young (left) and old (right) splenic ECs and immune cells which sent from Immunology2 EC.

(C) Circle network diagram showing the cell-cell interaction patterns in young (left) and old (right) splenic ECs and immune cells which sent from Immunology3 EC.

(D) Circle network diagram showing the cell-cell interaction patterns in young (left) and old (right) splenic ECs and immune cells which sent from Immunology4 EC.

(E) Dotplot representing the ligand-receptor pair between splenic ECs and immune cells (young and old intergrated), which splenic ECs are set as sender. The specific interaction between splenic EC and immune cells (Imm1-DC; Imm2-MC; Imm4-Macrophage) were labeled in red.

(F) Dotplot representing the ligand-receptor pair of specific interaction between splenic ECs and immune cells (Imm1-DC; Imm2-MC; Imm4-Macrophage). The young groups are labeled in coral and the old are labeled in cyan.

(G)Violin plot showing the gene expression of the indicated pathways between splenic ECs and immune cells.

**Additional file 4**：Table S1

List of the classification markers(top 50) for all cell subpopulations.

**Additional file 5**：Table S2

List of the Differential expression genes in all cell subpopulations.

**Additional file 6**：Table S3

List of the gene sets that used for Gene set score analysis. The source of gene sets can be found in Methods.

**Additional file 7**：Figure S4


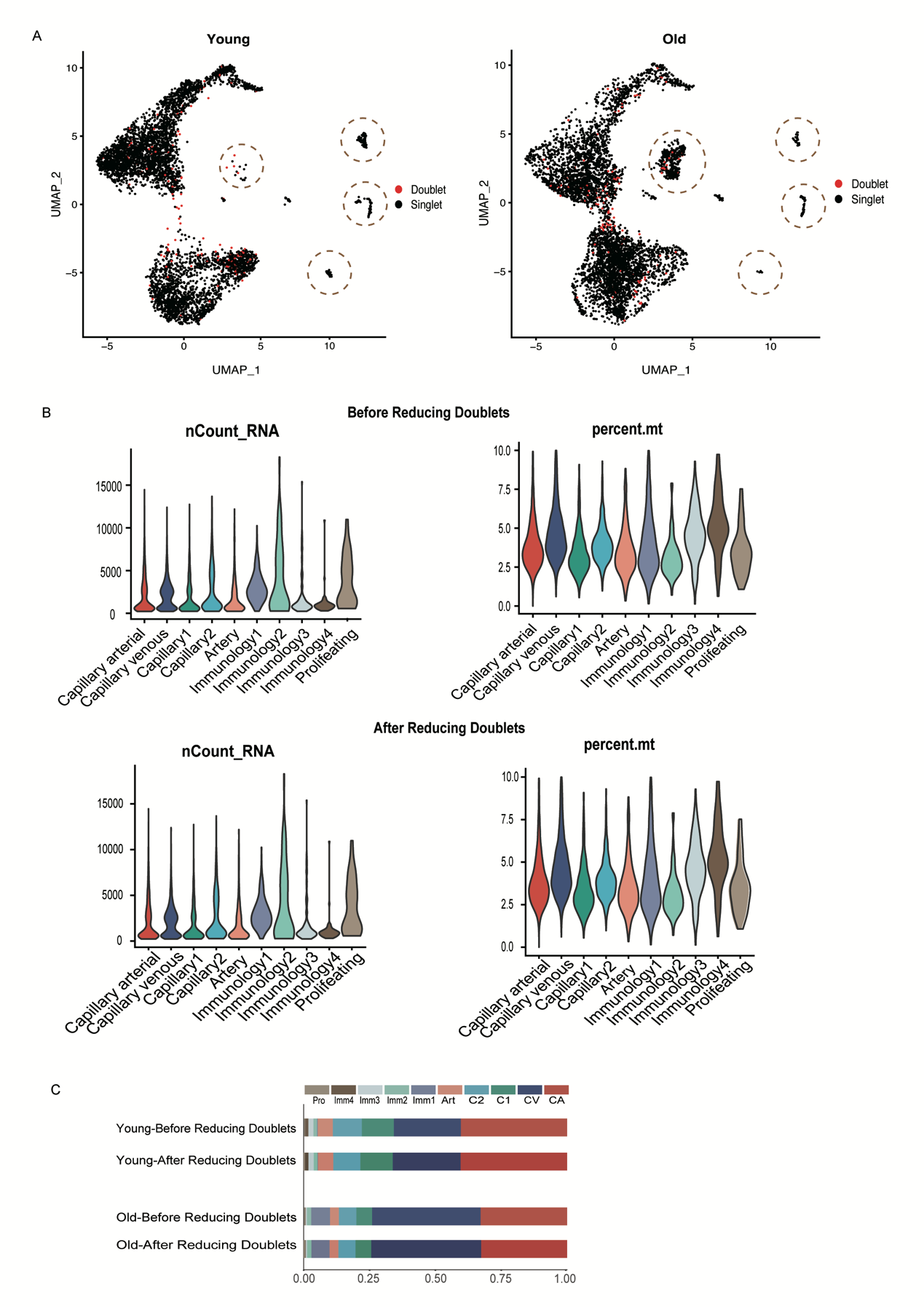


**Figure S4:Quality control and doublets identification in splenic young and old ECs**

1. Distribution of singlets and doublets ECs in the UMAP plots of young and old mouse. Four immunology-related clusters were circlized.
2. Violin plots showing the UMI in each celltype and the mitochondrial proportion before and after removing the doublets.
3. Barplot showing the proportion of celltype before and after removing the doublets.

**Additional file 8**：Figure S5


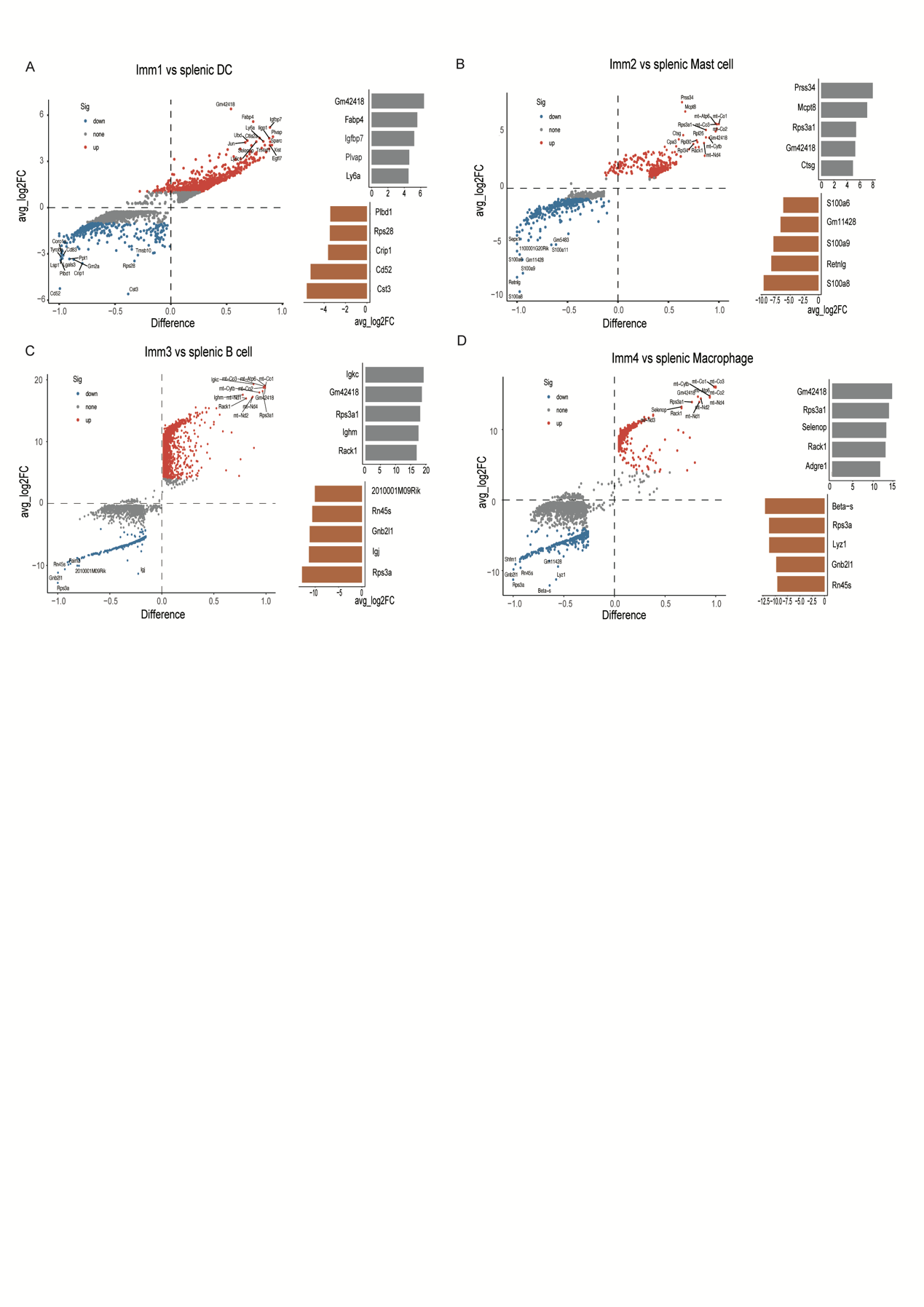


**Figure S5:DEGs between Immunology-related EC clusters and splenic immune cells**

1. Volcano plot showing the DEGs between Immunology1 and splenic DCs. Right: Barplot showing the top 5 DEGs in Immunology1 and splenic DCs.
2. Volcano plot showing the DEGs between Immunology2 and splenic Mast cells.Right: Barplot showing the top 5 DEGs in Immunology2 and splenic Mast cells.
3. Volcano plot showing the DEGs between Immunology3 and splenic B cells. Right: Barplot showing the top 5 DEGs in Immunology3 and splenic B cell.
4. Volcano plot showing the DEGs between Immunology4 and splenic Macrophages. Right: Barplot showing the top 5 DEGs in Immunology1 and splenic Macrophages.

**Additional file 9**：Figure S6


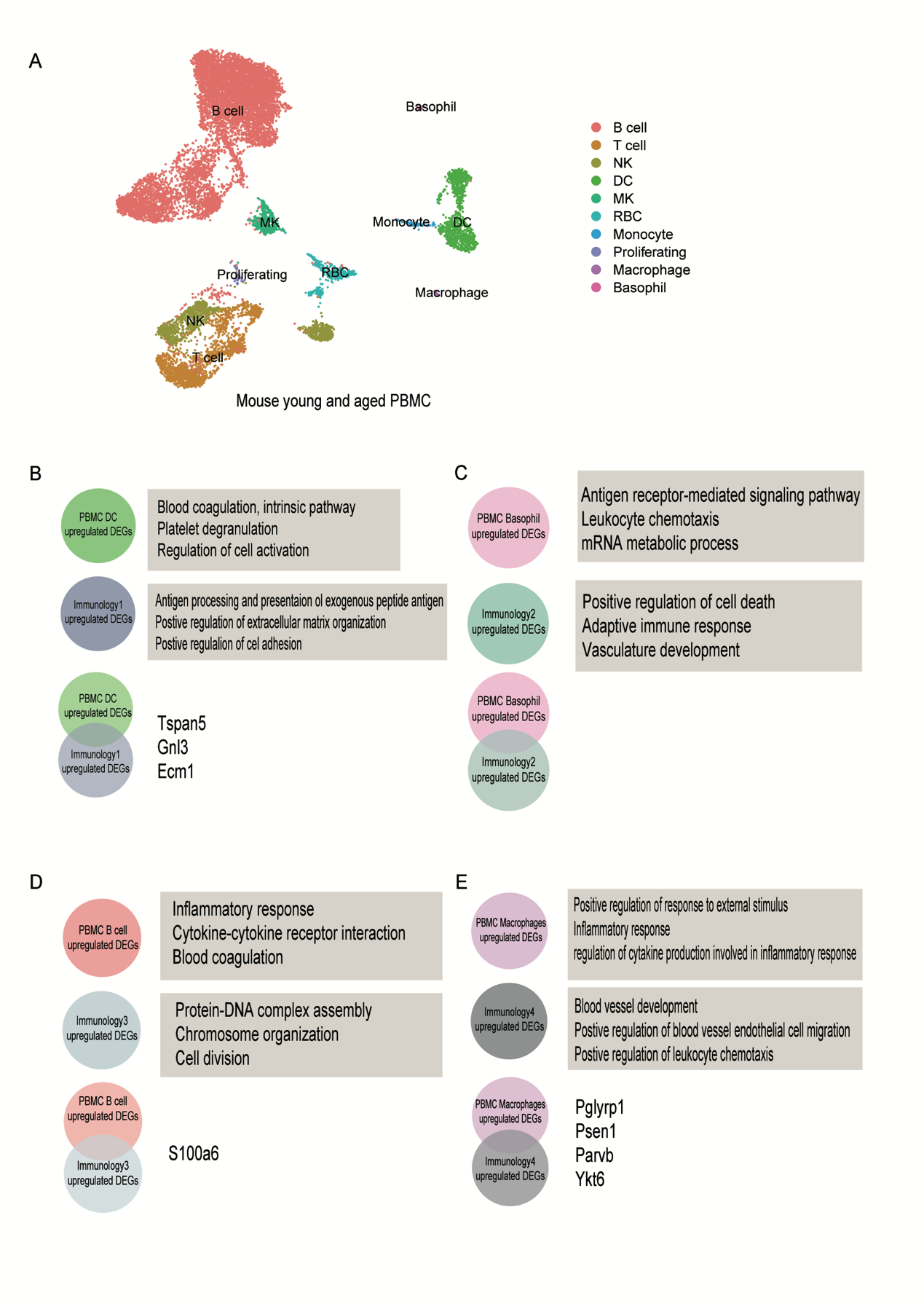


**Figure S6:The comparison of the aged-related changes between immunology-related ECs cluster and peripheral blood immune cells**

1. UMAP plot showing distribution of young and old mouse pbmc clusters.
2. The top 3 GO enrichment of PBMC DC and Immunology1 and the overlap upregulated DEGs**.**
3. The top 3 GO enrichment of PBMC Basophil and Immunology2 and the overlap upregulated DEGs**.**
4. The top 3 GO enrichment of PBMC B cell and Immunology3 and the overlap upregulated DEGs
5. The top 3 GO enrichment of PBMC Macrophage and Immunology4 and the overlap upregulated DEGs.

**Additional file 10**：Figure S7


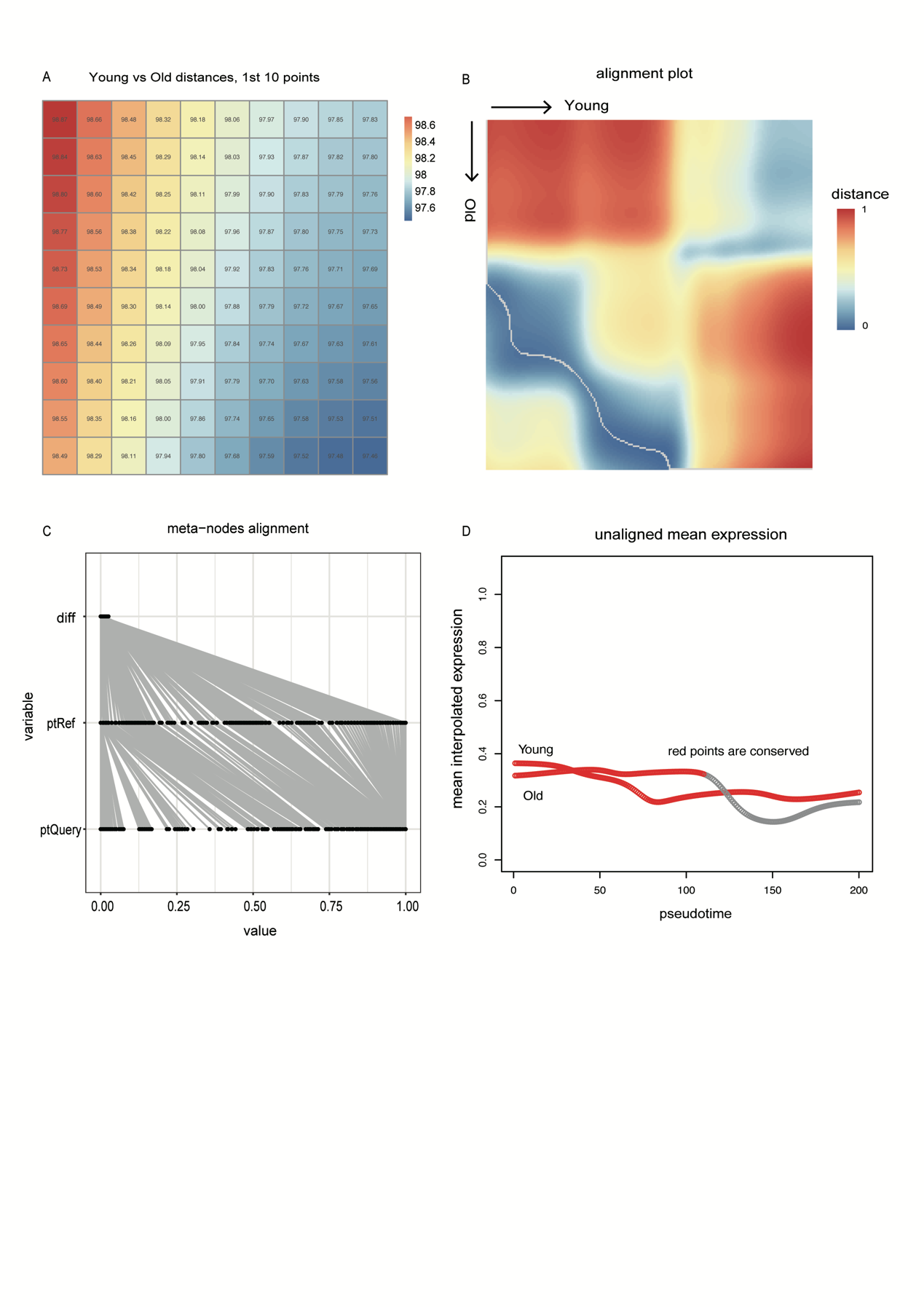


**Figure S7:Global and local alignment trajectory between young and old immunology4 subset**

1. Heatmap showing the distance between young and old immunology4 subset.
2. Alignment plot showing the global alignment trajectory between young and old immunology4 subset
3. Global-alignment-derived matchings between young and old immunology4 subset.
4. The pseudotime trajectory showing the expression of gene-module whose expression pattern is conserved early in young and old immunology4 subset and deviates afterwards. Values correspond to the median interpolated scaled expression across module genes. Locally aligned points are red, whereas unaligned, non-conserved points in the trajectory are gray

**Additional file 11**：Figure S8


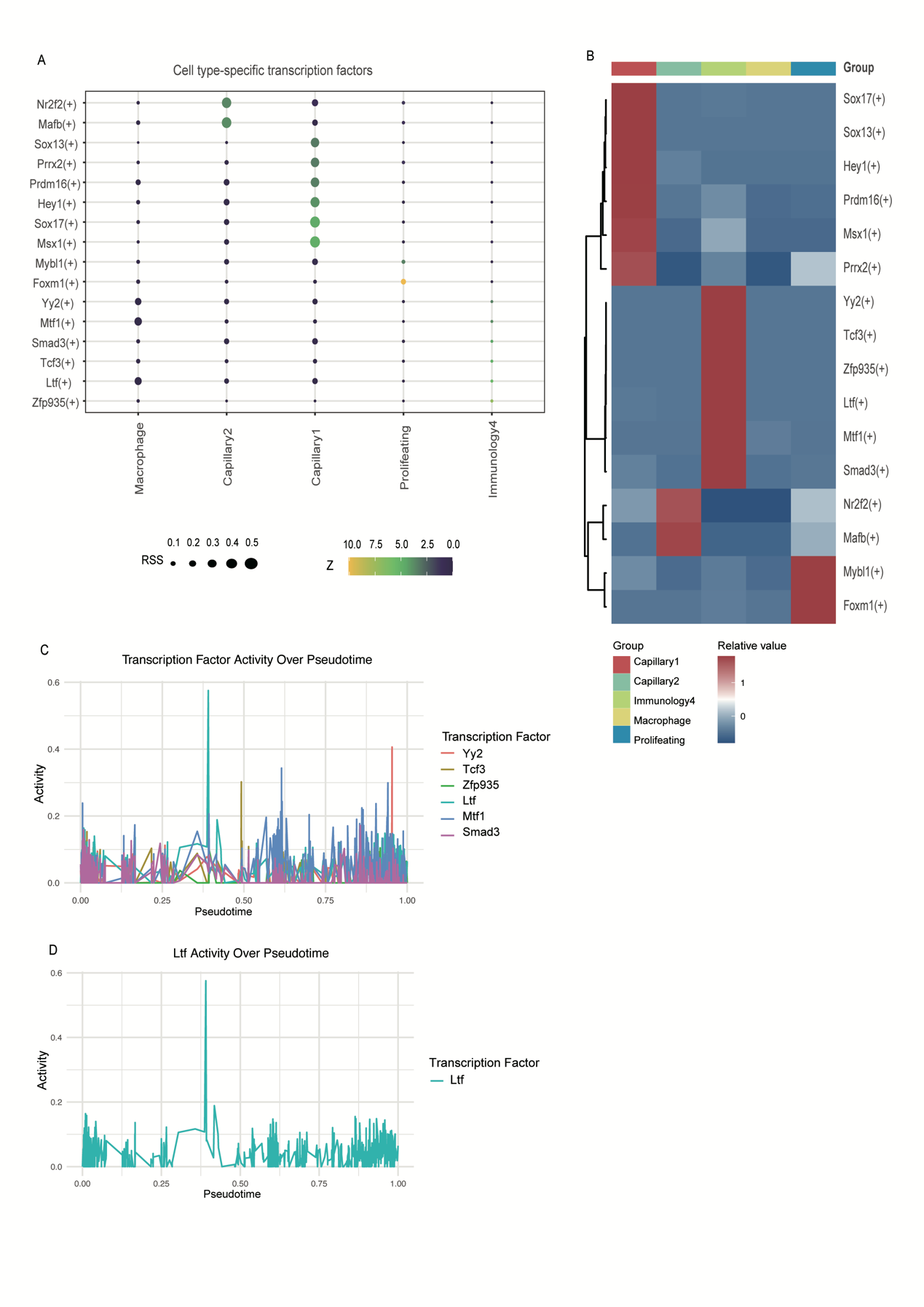


**Figure S8:Transcription factor of the old immunology4 subset along the pseudotime**

1. Dot plot showing the celltype-specific transcription factors in old immunology4 subset.
2. Heatmap showing the celltype-specific transcription in old immunology4 subset, the high relative values were colored in red while the low ones were colored in blue.
3. The curve plot showing the trajectory of immunology4-related transcription factors throughout the pseudotime
4. The curve plot showing the trajectory of Ltf throughout the pseudotime

**Additional file 12**：Figure S9


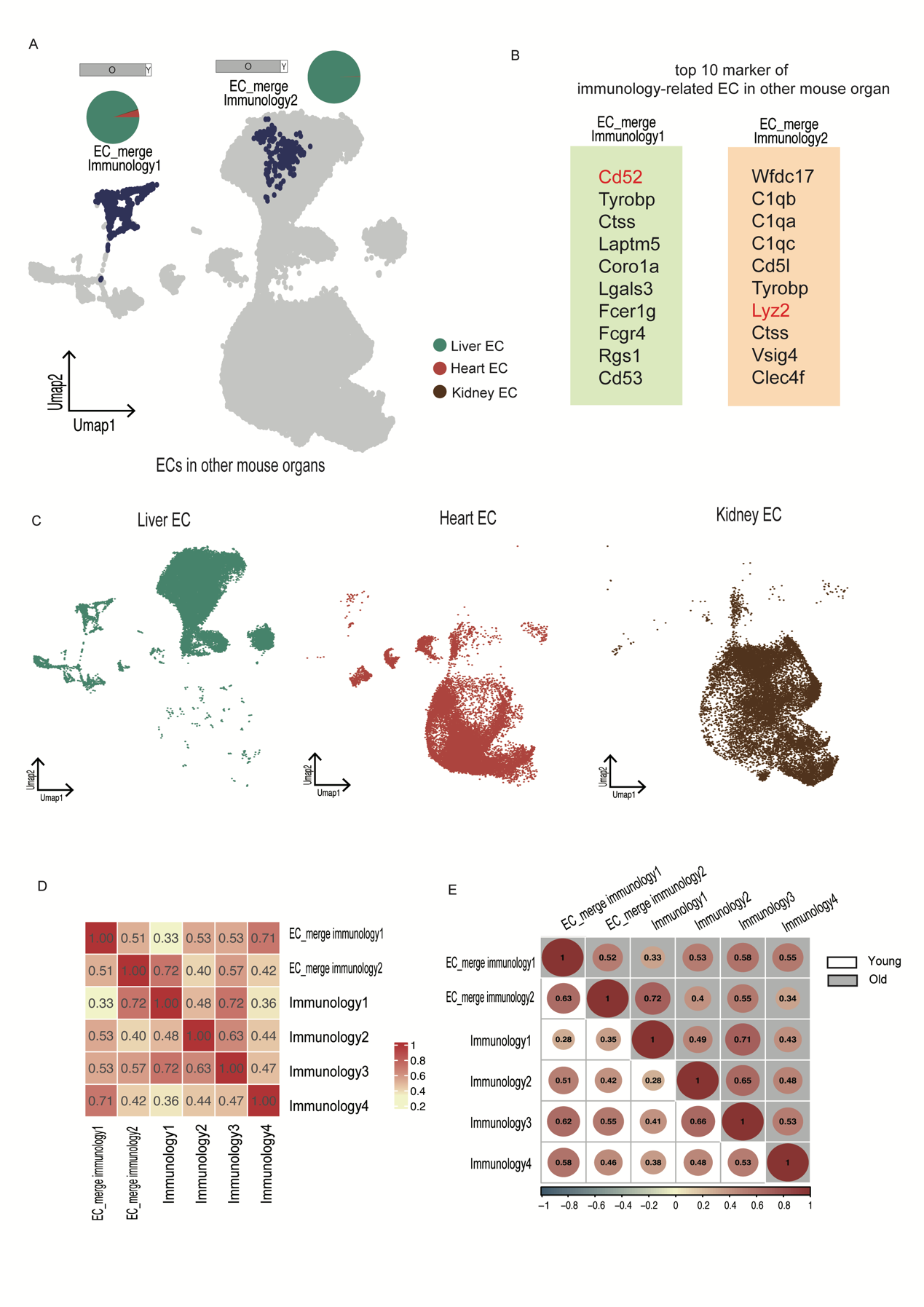


**Figure S9:Identification of the immunology-related ECs in other mouse organs**

1. UMAP plot showing intergrated ECs in different mouse organs, the immunology-related ECs were colored in navy. The barplots showing the rate of young and old groups while the pie chart showing the proportion of different organs.
2. Top 10 markers of immunology-related ECs in other mouse organs.
3. UMAP plot illustrating the distribution of ECs from mouse liver, kidney, and heart.
4. Correlation matrix displaying the relationships of immunology-related ECs between spleen and other organs.
5. Correlation matrix displaying the relationships between immunology-related ECs between spleen and other organs.The upper matrix, shaded in grey, illustrates the correlations in old group, while the lower matrix depicts the correlations for young ones. Each correlation coefficient is labeled within the corresponding circle in the matrix.

**Additional file 13**：Figure S10


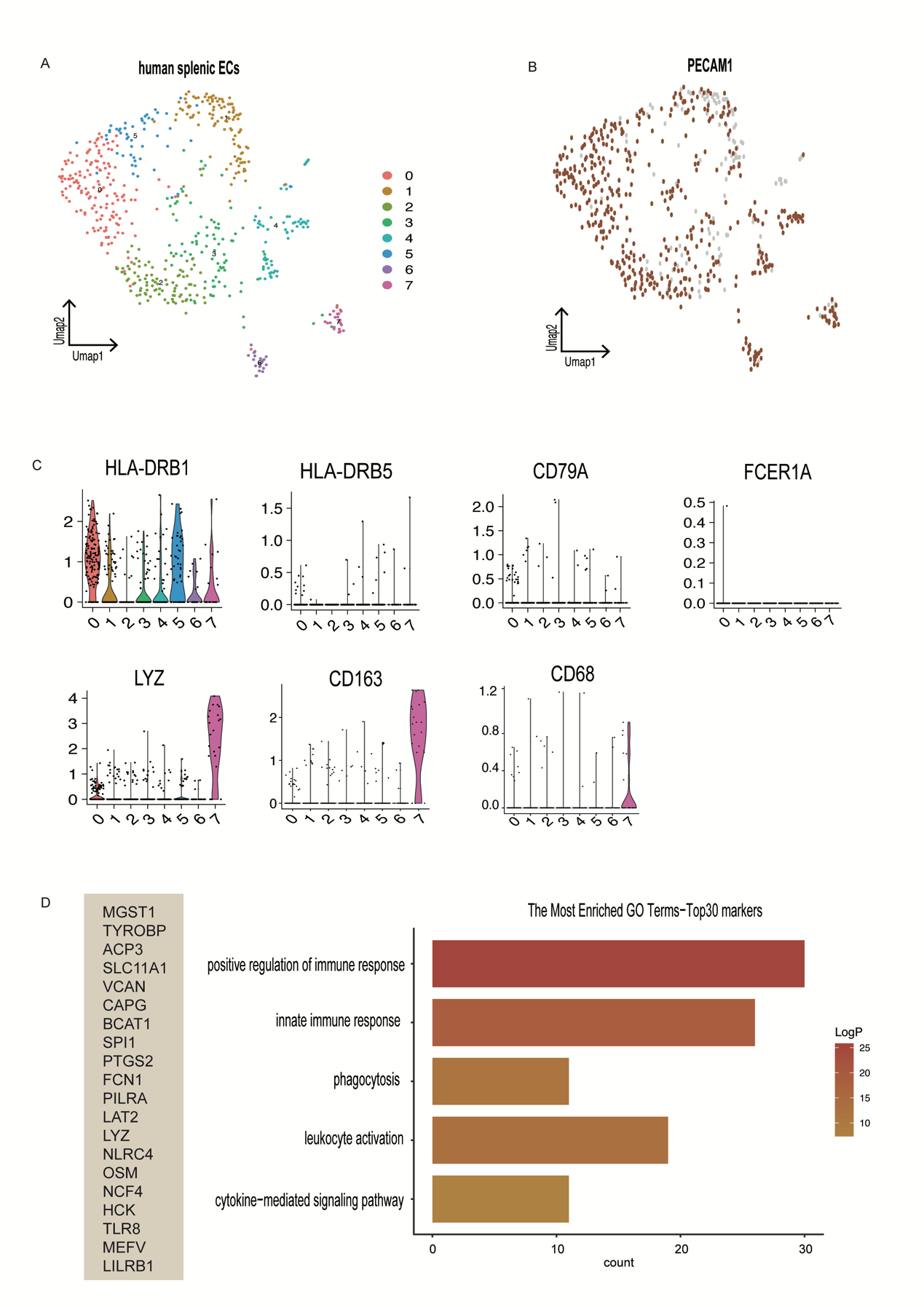


**Figure S10:Identification of immunology-related cluster in human splenic ECs**

1. UMAP plot showing the distribution of human splenic ECs
2. Feature plot showing the expression of PECAM1(CD31) in human splenic ECs.
3. Violin plot showing the expression of HLA-DRB1, HLA-DRB5, CD79A, FCER1A, LYZ, CD163, CD68 in each cluster of human splenic ECs.
4. Top 30 markers and the GO enrichment of the immunology-related EC in human spleen.
